# SGLT2 inhibitors and lower limb complications: an updated meta‐analysis

**DOI:** 10.1186/s12933-021-01276-9

**Published:** 2021-04-28

**Authors:** Chu Lin, Xingyun Zhu, Xiaoling Cai, Wenjia Yang, Fang Lv, Lin Nie, Linong Ji

**Affiliations:** 1 Department of Endocrinology and Metabolism, Peking University People’s Hospital, Beijing, China; 2 Department of Endocrinology and Metabolism, Beijing Airport Hospital, Beijing, China

**Keywords:** Sodium glucose co‐transporter 2 inhibitor, Blood pressure lowering agent, Peripheral arterial disease, Amputation, Diabetic foot, Diabetes mellitus

## Abstract

**Background:**

To exam the associations between the use of sodium glucose co-transporter 2 inhibitor (SGLT2i) and the risk of lower limb complications, and to analyze the associated factors.

**Methods:**

Pubmed, Medline, Embase, the Cochrane Center Register of Controlled Trials for Studies and *Clinicaltrial.gov* were searched from the inception to November 2020. Randomized controlled trials of SGLT2i conducted in population containing diabetic patients with reports of amputation, peripheral arterial disease (PAD) and diabetic foot (DF) events were included. Random-effect model, fixed-effect model and meta-regression analysis were accordingly used.

**Result:**

The numbers of SGLT2i users versus non-SGLT2i users in the analyses of amputation, PAD and DF were 40,925/33,414, 36,446/28,685 and 31,907/25,570 respectively. Compared with non-SGLT2i users, the risks of amputation and PAD were slightly increased in patients with canagliflozin treatment (amputation: OR = 1.60, 95% CI 1.04 to 2.46; PAD: OR = 1.53, 95 % CI 1.14 to 2.05). Meta-regression analyses indicated that greater weight reduction in SGLT2i users was significantly associated with the increased risks of amputation (β = − 0.461, 95% CI − 0.726 to − 0.197), PAD (β = − 0.359, 95% CI − 0.545 to − 0.172) and DF (β = − 0.476, 95% CI − 0.836 to − 0.116). Lower baseline diastolic blood pressure (β = − 0.528, 95% CI − 0.852 to − 0.205), more systolic blood pressure reduction (β = − 0.207, 95% CI − 0.390 to − 0.023) and more diastolic blood pressure reduction (β = − 0.312, 95% CI − 0.610 to − 0.015) were significantly associated with the increased risks of amputation, PAD and DF respectively in patients with SGLT2i treatment.

**Conclusions:**

The risks of amputation and PAD were slightly increased in patients with canagliflozin treatment. Reductions in body weight and blood pressure were associated with lower limb complications in patients with SGLT2i treatment.

## Background

Sodium-glucose cotransporter 2 inhibitor (SGLT2i) is an effective hypoglycemic agent in the management of type 2 diabetes (T2D), with significant improvements in glycemic control and favorable cardiovascular/renal benefits [1]. However, since almost two-fold incidence of amputation was reported in patients with canagliflozin treatment in CANVAS program, the United States Food and Drug Administration (FDA) issued a Boxed Warning to canagliflozin drug label [2, 3]. Although the Boxed Warning to the risk of amputation with canagliflozin has been encouragingly removed by FDA in the late August 2020, such unexpected concern still cast a shade on SGLT2i treatment in patients at high risk of lower limb complications [4]. As FDA suggested, despite the risk of amputation might be lower than previously described, a close monitoring is still needed to guarantee the safe application of canagliflozin [4].

Recently, several systematic reviews and meta-analyses have re-evaluated the association between SGLT2i and amputation, which indicated that SGLT2i was not associated with the increased risk of amputation [5, 6]. However, does it mean that we are in no worries to embrace the promoted applications of SGLT2i? It is noted that even though the Box Warning has been removed, the potential risk of major amputation with canagliflozin in patients with advanced atherosclerosis and impaired peripheral circulation still exists [4]. A large cohort study by using US database claimed that the increase in the rate of amputation with canagliflozin was most apparent among adults aged 65 or older with baseline cardiovascular disease [7]. As is known, the majority of lower limb amputation in patients with diabetes was due to uncontrolled infection and subsequent necrosis of extremities, which may be associated with other relevant conditions including peripheral arterial disease (PAD) and diabetic foot (DF) [8]. Such conditions may precede the surgical procedures and eventually lead to amputation. So far, the evaluations of PAD and DF are less characterized than amputation in patients with SGLT2i treatment.

Moreover, the explanations for the increased risk of amputation observed in patients with canagliflozin treatment are not definite. One hypothesis emphasized the hemodynamic effects of diuresis and blood pressure reduction within canagliflozin treatment. Similar effects were also found in diuretics, which might worsen the peripheral perfusion and in turn increase the risk of lower limb amputation in patients with T2D [9]. However, such scientific assumption has not been fully tested experimentally and clinically.

Therefore, we designed and performed an updated meta-analysis using data from randomized controlled trials (RCTs) of SGLT2i conducted in population containing diabetic patients, to hopefully produce a comprehensive safety evaluation on amputation, PAD and DF in patients using SGLT2i, and more importantly, to assess the association between altered hemodynamic status (diuresis and blood pressure reduction) and the risk of lower limb complications in SGLT2i users. Our study aims to provide state-of-the-art evidence-based guidance for appropriate clinical application of SGLT2i especially in patients at high risk of lower limb complications.

## Methods

### Data sources and searches

According to recommendations from the Cochrane Handbook for Systematic Reviews for meta-analysis, two independent investigators (NL and XZ) conducted systematic searches respectively for RCTs of SGLT2i in Pubmed, Medline, Embase, the Cochrane Center Register of Controlled Trials for Studies and *Clinicaltrial.gov* from the inception to November 2020. The search terms were as follows: type 1 diabetes (T1D); T2D; SGLT2i; cardiovascular outcome trial (CVOT); renal outcome trial (ROT); efficacy; safety; placebo controlled; RCT. The search strategies were as follows: (1) “T1D or T2D” and “CVOT or ROT” and “SGLT2i”; (2) “T1D or T2D” and “efficacy or safety” and “SGLT2i” and “RCT”; (3) “T1D or T2D” and “placebo controlled” and “SGLT2i” and “RCT”. The searching results from two investigators were checked together to form the final database for study selection. Reference lists of relevant articles were also screened in order not to miss any possibly eligible study.

### Study selection

The inclusion criteria for eligible studies were as follows: (1) RCTs conducted in population containing diabetic patients; (2) RCTs with reports of PAD, amputation and DF events; (3) RCTs with SGLT2i and placebo or non-SGLT2i active control in different treatment arms. The exclusion criteria for the meta-analysis for SGLT2i treatment were as follows: (1) RCTs without diabetic patients at all; (2) RCTs without reports of PAD, amputation and DF event.

### Data extraction and quality assessment

Two investigators (NL and XZ) abstracted data of all studies, including the publication infomation, study design, treatment arms, study duration, baseline characteristics, efficacy endpoints such as HbA1c change, weight change and blood pressure change etc., lower limb complication event, and assessed the qualities of the included studies. Another two investigators (CL and WY) checked the abstractions and assessments for accuracy. The risk of bias was evaluated using the Cochrane risk of bias tool. PAD, amputation and DF events would be extracted from *clinicaltrial.gov* website with unique registered RCT number if the data was absent in both articles and supplementary materials. PAD is defined as the narrowing or blockage of the vessels that carry blood from the heart to the legs. The pre-specific PAD events included in this meta-analysis were as follows: arterial thrombosis limb, arterial stenosis limb, ischemic limb pain, iliac artery stenosis, iliac artery occlusion, iliac artery embolism, femoral artery occlusion, femoral arterial stenosis, extremity necrosis, intermittent claudication, peripheral arterial occlusive disease, peripheral artery occlusion, peripheral artery ischemia, peripheral artery stenosis, peripheral artery thrombosis, peripheral artery circulatory failure and leg ischemia. We addressed discrepancies by inviting a third investigator (FL) to join the discussion, and resolved disagreements by consensus.

### Data synthesis and analysis

Continuous outcomes were evaluated by computing the weighted mean differences (WMDs) and 95% confidence intervals (CIs). Categorical outcomes were evaluated by computing the odd ratios (ORs) and accompanying 95% CIs. Higgins I^2^ statistics were used to evaluate the between-study heterogeneity, when an I^2^ value > 50% indicating high level of heterogeneity. Fixed-effects model was used for low level of heterogeneity and random-effects model was used for high level of heterogeneity. Meta-regression analyses were performed to evaluate the associations between different potential associated factors and the risks of PAD, amputation and DF. Publication bias was assessed via funnel plot tests. Statistical significance was considered at P < 0.05.

Statistical analyses were primarily performed by using the STATA statistical software package (version 11.0, Stata Corp, College Station, Texas, USA) and the Review Manager statistical software package (version 5.3, Nordic Cochrane Centre, Copenhagen, Denmark). Analyses were conducted according to the Preferred Reporting Items for Systematic Reviews and Meta-Analyses (PRISMA) guidelines for conducting and reporting meta-analyses of RCTs.

## Results

### Included studies

In total, there were 39 RCTs of SGLT2i treatment included in our study, among which 31 RCTs, 15 RCTs and 19 RCTs were included in the analyses for PAD, amputation and DF respectively (Additional file 1: Figure S1). The numbers of SGLT2i users versus non-SGLT2i users in the analyses of amputation, PAD and DF were 40,925/33,414, 36,446/28,685 and 31,907/25,570 respectively. The majority of enrolled RCTs with SGLT2i treatment were conducted in patients with diabetes, with 33 RCTs in T2D, 4 RCTs in T1D and 3 RCTs in mixed population of diabetic and non-diabetic patients. The baseline characteristics of all included RCTs were systematically summarized in Additional file 1: Table S1.

The risk of bias was evaluated by the Cochrane instrument, which suggested the overall risk of bias and the selective reporting was low (Additional file 1: Table S2). The publication bias was accessed by funnel plot test, which all showed even distributions (Additional file 1: Figures S2–S4).

### Risk of amputation, PAD and DF in patients with SGLT2i treatment

**Fig. 1:**
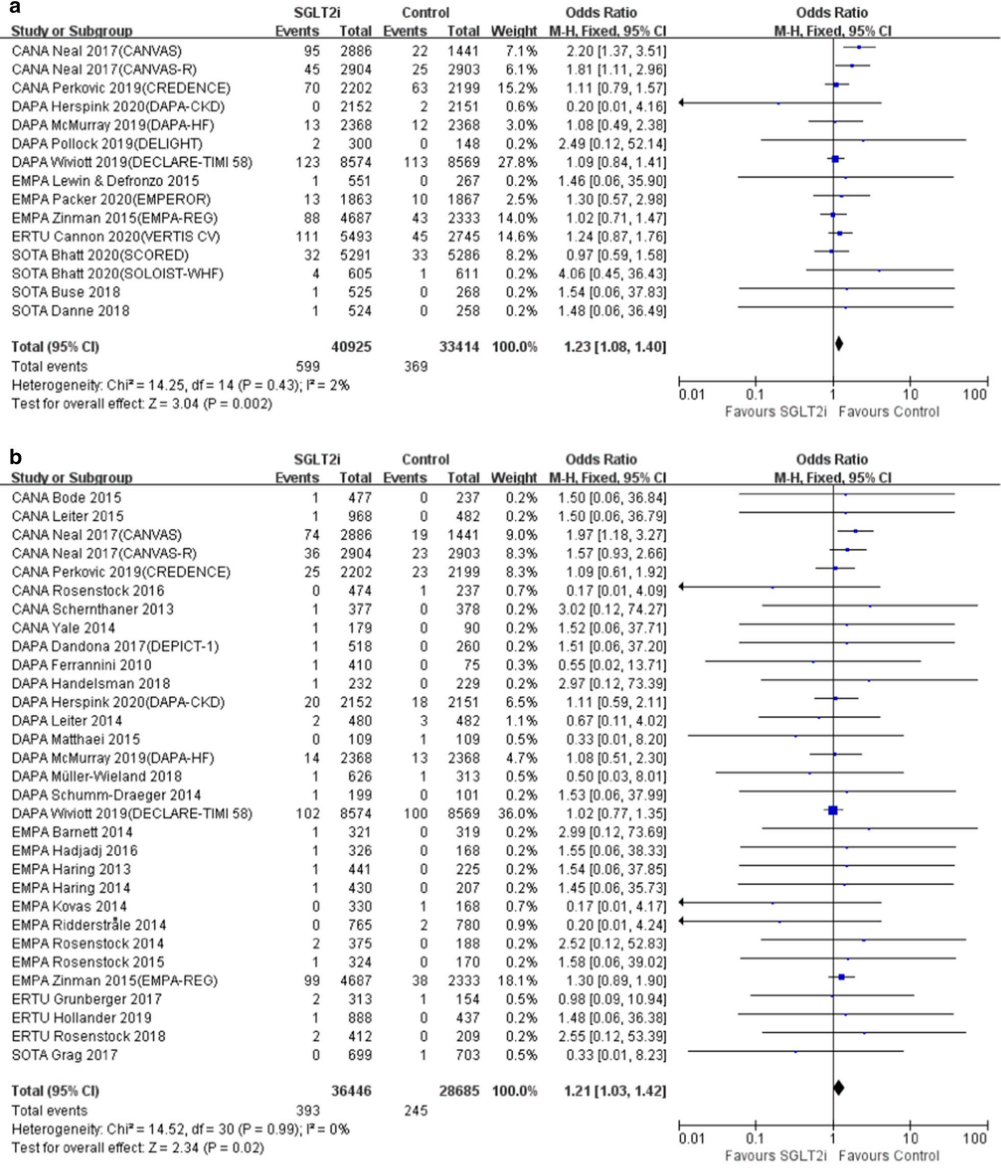
The risk of amputation and PAD in patients with SGLT2i treatment. **a** The risk of amputation in patients with SGLT2i treatment. **b** The risk of PAD in patients with SGLT2i treatment. PAD, peripheral arterial disease; SGLT2i, sodium glucose co-transporter 2 inhibitor

Compared with non-SGLT2i users, the risk of amputation (OR = 1.23, 95% CI 1.08 to 1.40, P = 0.002) (Fig. 1a) and the risk of PAD (OR = 1.21, 95% CI 1.03 to 1.42, P = 0.02) (Fig. 1b) were slightly increased in patients with SGLT2i treatment, which were mainly driven by the results from CVOTs and ROTs (Table 1). The drug-type analysis indicated that the increased risk of amputation (OR = 1.60, 95% CI 1.04 to 2.46, P = 0.03) and the risk of PAD (OR = 1.53, 95% CI 1.14 to 2.05, P = 0.005) were only observed in canagliflozin subgroup but not in other subtypes of SGLT2i. As for study population, the incidence of amputation (OR = 1.24, 95% CI 1.08 to 1.42, P = 0.002) and PAD (OR = 1.23, 95% CI 1.03 to 1.45, P = 0.02) were significantly increased in SGLT2i users versus non-SGLT2i users, which was only observed in pure diabetic population strata (Table 1). Moreover, the risk of amputation (OR = 1.22, 95% CI 1.07 to 1.39, P = 0.02) and the risk of PAD (OR = 1.22, 95% CI 1.03 to 1.44, P = 0.02) were significantly higher in RCTs with study duration longer than 52 weeks, when compared with non-SGLT2i users (Table 1). However, the incidence of DF was not elevated in SGLT2i users, which remained quite consistent among multiple sensitivity analyses (Table 1).

**Table 1:** Risk of amputation, PAD and DF events in patients with SGLT2i treatment

Subgroup	No. of participants (SGLT2i/control)	OR	95% CI	P value	I^2^ (%)
Risk of amputation by SGLT2i subtypes					
In total*	40,925/33,414	1.23	1.08, 1.40	0.002	2
Canagliflozin*	7992/6543	1.60	1.04, 2.46	0.03	67
Dapagliflozin	13,394/13,236	1.08	0.85, 1.37	0.54	0
Empagliflozin	7101/4467	1.07	0.76, 1.49	0.71	0
Ertugliflozin	5493/2745	1.24	0.87, 1.76	0.23	NA
Sotagliflozin	6945/6423	1.08	0.68, 1.70	0.75	0
Risk of amputation by study types					
CVOT and ROT*	39,025/32,473	1.23	1.07, 1.40	0.003	29
Efficacy and safety evaluation	1900/941	1.74	0.36, 8.39	0.49	0
Risk of amputation by population					
DM only*	34,872/27,196	1.24	1.08, 1.42	0.002	14
Including patients without DM	6383/6386	1.08	0.62, 1.88	0.78	0
Risk of amputation by control types					
Active agent	551/267	1.46	0.06, 35.90	0.82	NA
Placebo*	40,374/33,147	1.23	1.08, 1.40	0.002	9
Risk of amputation by study duration					
< 26 weeks	300/148	2.49	0.12, 52.14	0.56	0
26–52 weeks	2205/1404	2.34	0.58, 9.52	0.23	0
> 52 weeks*	33,129/26,576	1.24	1.08, 1.43	0.002	33
Risk of PAD by SGLT2i subtypes					
In total*	36,446/28,685	1.21	1.03, 1.42	0.02	0
Canagliflozin*	10,467/7967	1.53	1.14, 2.05	0.005	0
Dapagliflozin	15,668/14,657	1.03	0.81, 1.30	0.83	0
Empagliflozin	7999/4558	1.26	0.88, 1.78	0.20	0
Ertugliflozin	1613/800	1.50	0.30, 7.43	0.62	0
Sotagliflozin	699/703	0.33	0.01, 8.23	0.50	NA
Risk of PAD by study types					
CVOT and ROT*	25,773/21,964	1.24	1.05, 1.46	0.01	6
Efficacy and safety evaluation	10,673/6721	0.96	0.55, 1.68	0.90	0
Risk of PAD by population					
DM only*	31,926/24,166	1.23	1.03, 1.45	0.02	0
Including patients without DM	4520/4519	1.10	0.67, 1.79	0.71	0
Risk of PAD by control types					
Active agent	3856/2619	1.00	0.33, 3.06	1.00	0
Placebo*	32,590/26,066	1.22	1.03, 1.43	0.02	0
Risk of PAD by study duration					
< 26 weeks	5043/3159	0.91	0.43, 1.90	0.80	0
26–52 weeks	2766/1725	1.70	0.50, 5.76	0.40	0
> 52 weeks*	28,637/23,801	1.22	1.03, 1.44	0.02	0
Risk of DF by SGLT2i subtypes					
In total	31,907/25,570	1.24	0.93, 1.64	0.14	0
Canagliflozin	9139/7115	1.55	0.94, 2.54	0.09	0
Dapagliflozin	14,638/13,809	1.20	0.79, 1.83	0.40	0
Empagliflozin	7029/4055	0.90	0.48, 1.67	0.74	0
Ertugliflozin	1201/591	1.48	0.15, 14.28	0.73	0
Risk of DF by study types					
CVOT and ROT	25,773/21,964	1.23	0.91, 1.66	0.17	0
Efficacy and safety evaluation	6134/3606	1.26	0.55, 2.92	0.59	0
Risk of DF by population					
DM only	27,387/21,051	1.28	0.95, 1.72	0.11	0
Including patients without DM	4520/4519	0.89	0.34, 2.31	0.81	0
Risk of DF by control types					
Active agent	4173/2467	1.54	0.44, 5.34	0.50	0
Placebo	27,734/23,103	1.22	0.91, 1.63	0.18	0
Risk of DF by study duration					
< 26 weeks	1131/562	1.49	0.23, 9.51	0.67	0
26–52 weeks	2940/1614	1.51	0.44, 5.16	0.51	0
> 52 weeks	27,836/23,394	1.22	0.91, 1.63	0.19	0

*P < 0.05

*PAD *peripheral arterial disease, *SGLT2i *sodium glucose co-transporter 2 inhibitor, *DF* diabetic foot, *DM *diabetes mellitus, *CVOT *cardiovascular outcome trial, *ROT *renal outcome trial, *OR *odd ratio, *CI *confidence interval, *NA *not applicable

### Associated factors with the risk of amputation, PAD and DF in SGLT2i treatment

**Fig. 2:**
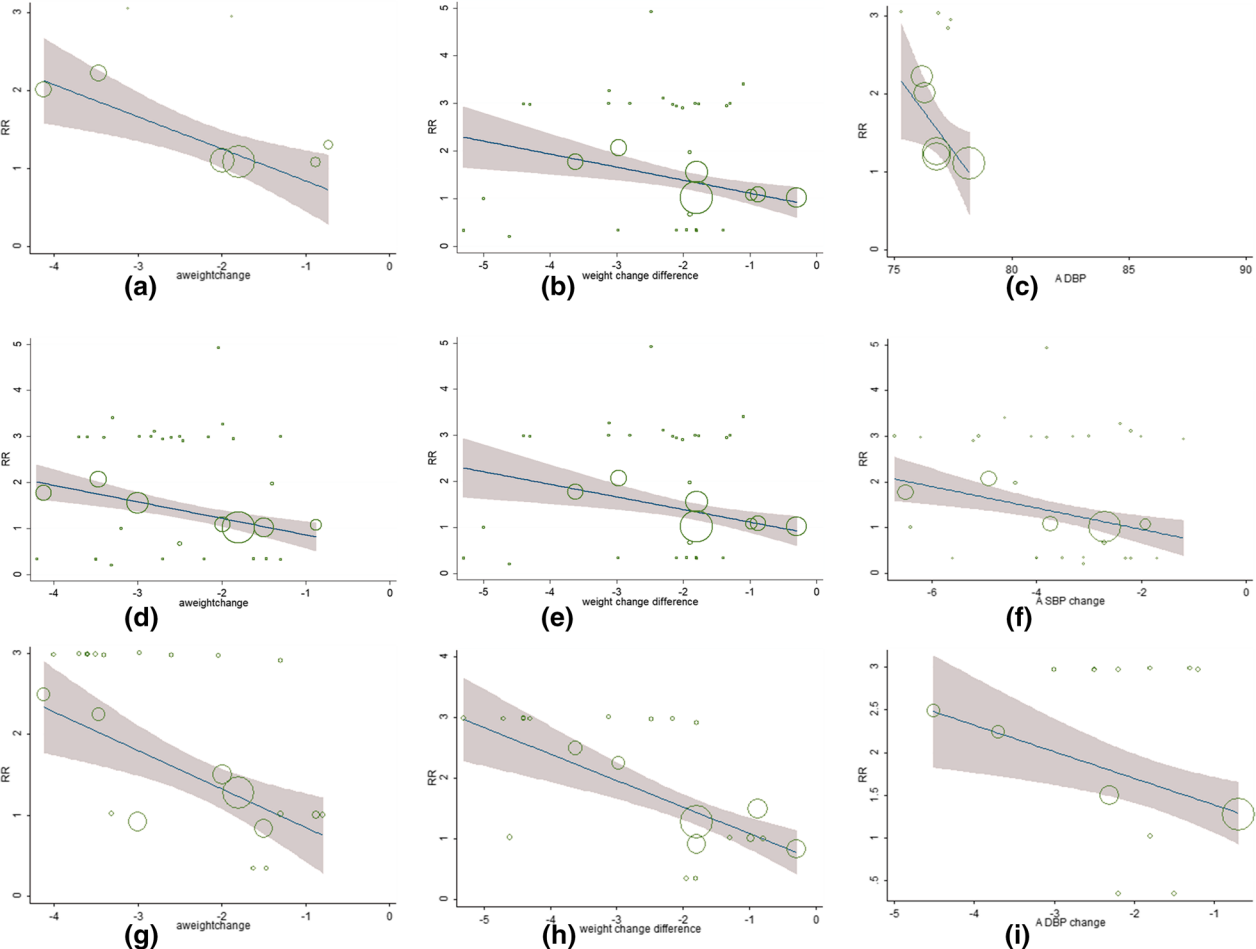
Meta-regression analysis for the associated factors and risk of lower limb complications in patients with SGLT2i treatment. **a** The association between weigh change and risk of amputation in patients with SGLT2i treatment. **b** The association between weigh change difference and risk of amputation in patients with SGLT2i treatment. **c** The association between baseline DBP and risk of amputation in patients with SGLT2i treatment. **d** The association between weigh change and risk of PAD in patients with SGLT2i treatment. **e** The association between weigh change difference and risk of PAD in patients with SGLT2i treatment. **f** The association between SBP change and risk of PAD in patients with SGLT2i treatment. **g** The association between weigh change and risk of DF in patients with SGLT2i treatment. **h** The association between weigh change difference and risk of DF in patients with SGLT2i treatment. **i** The association between DBP change and risk of DF in patients with SGLT2i treatment. DBP, diastolic blood pressure; DF, diabetic foot; PAD, peripheral arterial disease; SBP, systolic blood pressure; SGLT2i, sodium glucose co-transporter 2 inhibitor

According to the results of meta-regression analyses, greater weight reduction in SGLT2i users was significantly associated with the risk of amputation (β = − 0.461, 95% CI − 0.726 to − 0.197, P = 0.007), the risk of PAD (β = − 0.359, 95% CI − 0.545 to − 0.172, P < 0.001) and the risk of DF (β = − 0.476, 95% CI − 0.836 to − 0.116, P = 0.012) (Fig. 2; Tables 2, 3 and 4). Consistently, greater weight reduction difference between SGLT2i and control groups was also significantly associated with the risk of amputation (β = − 0.436, 95% CI − 0.795 to − 0.078, P = 0.026), the risk of PAD (β = − 0.264, 95% CI − 0.472 to − 0.056, P = 0.014) and the risk of DF (β = − 0.437, 95% CI  − 0.719 to − 0.155, P = 0.004) in SGLT2i users (Fig. 2; Tables 2, 3 and 4). Moreover, lower baseline diastolic blood pressure (β = − 0.528, 95% CI − 0.852 to − 0.205, P = 0.009), more systolic blood pressure reduction (β = − 0.207, 95% CI − 0.390 to − 0.023, P = 0.028) and more diastolic blood pressure reduction (β = − 0.312, 95% CI − 0.610 to − 0.015, P = 0.041) were significantly associated with increased risks of amputation, PAD and DF respectively in SGLT2i users (Fig. 2; Tables 2, 3 and 4). Other factors such as age, sex, body mass index, duration of diabetes, renal function, previous history of PAD, or HbA1c reduction achieved by SGLT2i, were not associated with the risk of lower limb complications (Tables 2, 3 and 4).

**Table 2:** Meta-regression analysis for the associated factors and risk of amputation events in patients with SGLT2i treatment

Parameter	β	95% CI	P value	Parameter	β	95% CI	P value
Demographic characteristic							
Age (year old)	− 0.090	− 0.224, 0.045	0.163	Sex (male%)	0.013	− 0.023, 0.049	0.780
BMI (kg/m^2^)	0.097	− 0.231, 0.426	0.506	Diabetes duration (year)	0.177	− 0.036, 0.390	0.984
HbA1c and weight change							
HbA1c change (%)	0.689	− 4.454, 5.832	0.761	Weight change (kg)*	− 0.461	− 0.726, − 0.197	0.007
HbA1c change difference (%)	− 0.479	− 6.171, 5.213	0.848	Weight change difference (kg)*	− 0.436	− 0.795, − 0.078	0.026
Hemodynamic indicator							
Baseline SBP (mmHg)	0.005	− 0.094, 0.105	0.902	Baseline DBP (mmHg)*	− 0.528	− 0.852, − 0.205	0.009
SBP change (mmHg)	− 0.254	− 0.545, 0.037	0.075	DBP change (mmHg)	− 0.279	− 0.638, 0.079	0.096
Volume depletion (event%)	− 0.372	− 0.900, 0.155	0.121	−	−	−	−
Renal function profile							
Baseline eGFR (mL/min/1.73m^2^)	0.058	− 0.393, 0.508	0.352	eGFR change (mL/min/1.73m^2^)	0.393	− 1.183, 1.969	0.550
PAD risk factor							
Previous PAD (%)	0.003	− 0.296, 0.301	0.973	−	−	−	−

*P < 0.05. *BMI *body mass index, *SGLT2i *sodium glucose co-transporter 2 inhibitor, *PAD *peripheral arterial disease, *SBP *systolic blood pressure, *DBP *diastolic blood pressure, *HCT *hematocrit, *eGFR* estimated glomerular filtration rate

**Table 3:** Meta-regression analysis for the associated factors and risk of PAD events in patients with SGLT2i treatment

Parameter	β	95% CI	P value	Parameter	β	95% CI	P value
Demographic characteristic							
Age (year old)	− 0.057	− 0.133, 0.018	0.133	Sex (male%)	− 0.003	− 0.026, 0.019	0.780
BMI (kg/m^2^)	− 0.011	− 0.071, 0.050	0.720	Diabetes duration (year)	− 0.001	− 0.104, 0.102	0.984
HbA1c and weight change							
HbA1c change (%)	− 0.540	− 1.518, 0.437	0.269	Weight change (kg)*	− 0.359	− 0.545, − 0.172	< 0.001
HbA1c change difference (%)	− 1.126	− 3.166, 0.914	0.269	Weight change difference (kg)*	− 0.264	− 0.472, − 0.056	0.014
Hemodynamic indicator							
Baseline SBP (mmHg)	0.004	− 0.061, 0.070	0.896	Baseline DBP (mmHg)	0.017	− 0.338, 0.372	0.922
SBP change (mmHg)*	− 0.207	− 0.390, − 0.023	**0.028**	DBP change (mmHg)	− 0.191	− 0.493, 0.110	0.201
Hemoglobin change (g/L)	− 0.076	− 0.746, 0.594	0.782	HCT change	− 0.242	− 1.117, 0.634	0.552
Volume depletion (event%)	− 0.061	− 0.269, 0.147	0.528	Osmotic diuresis (event%)	0.367	− 0.091, 0.825	0.091
Renal function profile							
Baseline eGFR (mL/min/1.73m^2^)	0.019	− 0.014, 0.051	0.242	eGFR change (mL/min/1.73m^2^)	− 0.184	− 0.473, 0.107	0.200
PAD risk factor							
Previous PAD (%)	0.017	− 0.039, 0.072	0.411	−	−	−	−

*P < 0.05. *BMI *body mass index, *SGLT2i *sodium glucose co-transporter 2 inhibitor, *PAD *peripheral arterial disease, *SBP *systolic blood pressure, *DBP *diastolic blood pressure, *HCT *hematocrit, *eGFR *estimated glomerular filtration rate

**Table 4:** Meta-regression analysis for the associated factors and risk of DF events in patients with SGLT2i treatment

Parameter	β	95% CI	P value	Parameter	β	95% CI	P value
Demographic characteristic							
Age (year old)	− 0.089	− 0.192, 0.014	0.087	Sex (male%)	− 0.021	− 0.058, 0.016	0.247
BMI (kg/m^2^)	− 0.001	− 0.060, 0.058	0.920	Diabetes duration (year)	− 0.027	− 0.187, 0.133	0.732
HbA1c and weight change							
HbA1c change (%)	− 1.723	− 3.749, 0.303	0.091	Weight change (kg)*	− 0.476	− 0.836, − 0.116	0.012
HbA1c change difference (%)	1.305	− 1.289, 3.900	0.307	Weight change difference (kg)*	− 0.437	− 0.719, − 0.155	0.004
Hemodynamic indicator							
Baseline SBP (mmHg)	0.023	− 0.085, 0.131	0.660	Baseline DBP (mmHg)	0.201	− 0.364, 0.767	0.458
SBP change (mmHg)	− 0.264	− 0.541, − 0.013	0.060	DBP change (mmHg)*	− 0.312	− 0.610, − 0.015	0.041
Hemoglobin change (g/L)	− 0.902	− 2.010, 0.206	0.087	HCT change	0.015	− 1.164, 1.193	0.976
Volume depletion (event%)	− 0.007	− 0.075, 0.061	0.772	−	−	−	−
Renal function profile							
Baseline eGFR (mL/min/1.73m^2^)	− 0.003	− 0.041, 0.035	0.867	eGFR change (mL/min/1.73m^2^)	− 0.114	− 0.755, 0.527	0.706
PAD risk factor							
Previous PAD (%)	− 0.007	− 0.075, 0.061	0.772	−	−	−	−

*P < 0.05. *BMI *body mass index, *SGLT2i *sodium glucose co-transporter 2 inhibitor, *PAD *peripheral arterial disease, *DF *diabetic foot, *SBP *systolic blood pressure, *DBP *diastolic blood pressure, *HCT *hematocrit, *eGFR *estimated glomerular filtration rate

## Discussion

By using the data from RCTs of SGLT2i, we found that the risks of amputation and PAD were slightly increased in patients using SGLT2i, mainly derived from the use of canagliflozin. But the use of SGLT2i did not increase the incidence of DF events. Furthermore, we found that the body weight reduction and blood pressure reduction in SGLT2i users were associated with the risks of amputation, PAD and DF, which might provide new insights and supporting evidence for the hemodynamic hypothesis and promote the evidence-based prevention of lower limb complications concerning the application of SGLT2i in the future. In addition to routine foot care, monitoring body weight and blood pressure change might be a useful precaution action for risk management during the SGLT2i treatment course, especially for patients at high risk of lower limb complications.

SGLT2i reduces plasma glucose by increasing the renal threshold for glucose reabsorption, leading to increased urinary glucose excretion and osmotic diuresis, which may be associated with a reduction in intravascular volume [10]. According to the results from this meta-analysis, we supposed that the reductions in body weight and blood pressure might indirectly reflect the body fluid loss and altered hemodynamic status in SGLT2i users. Reductions in body weight by SGLT2i are mainly mediated by two mechanisms: osmotic diuresis and urinary glucose excretion. Previous studies indicated that the early phase of weight reduction in SGLT2i users was caused by the enhanced diuresis and the increased loss of intravascular volume, since the adiposity volume was mildly changed up to first 4–8 weeks [11–13]. When excess body fluid was excreted and a dry body weight was reached, SGLT2i continued to reduce or maintain the body weight by urinary glucose excretion and additional regulatory effects on metabolism [14], which might play greater role in the later phase of weight reduction. Actually, an increased risk of lower extremity amputation was previously reported in patients with T2D using thiazide diuretics [9]. Diuretic-induced volume depletion might contribute to the circulatory failure in the distal peripheral arterial beds and consequently worsened the insufficient perfusion in the extremities. Therefore, similar diuretic effect of SGLT2i may also be associated with insufficient perfusion, which subsequently facilitates the development of lower limb complications in those susceptible patients.

Whether the weight reduction achieved through urinary glucose excretion with SGLT2i treatment plays a role in the risk of lower limb complications? To answer this question, another anti-diabetic agent, glucagon-like peptide-1 receptor analog (GLP-1RA), might give us the hints. GLP-1RA, enhances glucose-dependent insulin secretion, inhibits glucagon secretion, slows gastric emptying, and reduces food intake [15, 16]. Unlike SGLT2i, GLP-1RA leads to weight reduction mainly through energy intake restriction without diuretic effects. According to our previous meta-analysis of 97 RCTs, we proved that treatment of SGLT2i and GLP-1RA led to comparable weight changes from baseline, which were both with significance when compared with placebo treatment [17]. However, no reports of the increased risk of amputation events in patients with GLP-1RA treatment were observed, which is consistent with the observations in the real-world study [18]. Moreover, a nationwide register-based cohort study indicated that the use of SGLT2i, when compared with GLP-1RA, was associated with an increased risk of lower limb amputation (incidence rate 2.7 vs. 1.1 events per 1000-person years, hazard ratio 2.32, 95% CI 1.37 to 3.91) [19]. Therefore, it could be assumed that simply weight reduction through energy restrictions might not increase the risk of lower limb amputation. However, the interactions of diuresis and urinary glucose excretion might additively amplify the transient hemodynamic instability, which might be associated with increased risk of amputation and PAD in patients with SGLT2i treatments. But still, apart from GLP-1RA, the associations between other weight-reducing drugs such as orlistat and risk of lower limb complications remains uncertain and requires further investigations.

Blood pressure is another indicator for the hemodynamic status. Our study showed that baseline diastolic blood pressure, greater systolic blood pressure reduction and greater diastolic blood pressure reduction were significantly associated with increased risks of amputation, PAD and DF respectively in SGLT2i users. Blood pressure, as an important vital sign, could also reflect the peripheral perfusion status. Greater blood pressure reduction could be a result of reduced intravascular volume. As is mentioned earlier, the use of thiazide diuretics might increase the risk of lower limb amputation in patients with T2D [9]. The anti-hypertensive mechanism of thiazide diuretics is to reduce water and sodium retention, which again suggested that the diuresis and blood pressure reduction might be associated with the increased risk of lower limb complications observed in these very patients. However, the evidence regarding other kinds of blood pressure lowering drugs and the risk of lower limb complications is so far limited. Whether the associations of the blood pressure lowering and the risk of lower limb complications that observed in thiazide diuretics may also apply to a broad spectrum of hypotensive agents remains to be evaluated. Moreover, our previous meta-analysis of 94 RCTs showed that systolic blood pressure reduction in patients with SGLT2i was significantly associated with weight reduction [20]. It indicates that continuous weight reduction in the later phase caused by loss of adiposity might also facilitate the blood pressure reduction in patients with SGLT2i treatment, which further interrupts the hemodynamic hemostasis in the peripheral circulation and in turn contributes to the increased risk of lower limb complications.

It is noted that SGLT2i may also increase diet and water intakes as compensatory feedbacks in response to glycosuria and diuresis. Compensatory increased sugar intake has been reported in patients with T2D under dapagliflozin treatment [21]. However, although daily sucrose intake significantly increased in patients taking dapagliflozin for 3 months, daily intakes of total calories and the proportions of the three major nutrients were not significantly increased in patients either with or without dapagliflozin treatment [21]. Another prospective study in 26 patients with T2D indicated that dapagliflozin resulted in a significantly reduction in body weight by 12 weeks without inducing prominent hyperphagia [22]. As is mentioned before, the diuresis-related weight reduction may last for 4–8 weeks until a new homeostasis of body fluid is established through compensatory water intakes and other hormonal, metabolic and hemodynamic adaptions [11–13]. But during this time, the potential influence of early circulatory insufficiency may still occur. Actually, the weight-loss and blood-pressure-lowering effects have been observed and validated in multiple RCTs with long-term use of SGLT2i. It seems that the compensatory diet and water intakes might not be adequate to fully counteract the effects on body weight and blood pressure by SGLT2i-induced glycosuria and diuresis. In the sense, the reductions in body weight and blood pressure after SGLT2i treatment are still of clinical significance when it comes to lower limb complications in diabetes.

Based on the above analyses, we proposed that, in patients with high risk of lower limb complications, loss of intravascular volume at the early phase with SGLT2i treatment, reflected as diuresis-mediated weight reduction and blood pressure reduction, may contribute to first ischemic damage for the extremities in these patients. Continuous weight reduction at the later phase with SGLT2i treatment would further facilitate the blood pressure reduction and worsen the insufficient distal perfusion, which might contribute to the gradual development of PAD. Surgical removals might be eventually required in some extremely severe cases.

Furthermore, according to our meta-analysis, typical volume depletion indicators such as hematocrit, hemoglobin, were not associated with the increased risks of amputation and PAD. Such inconsistence might be due to the limited available data for the variables mentioned above. However, in patients with mild to moderate loss of body fluid, we assumed that weight reduction and blood pressure reduction rather than typical volume depletion manifestations, could still be observed. Thus, such subclinical volume depletion in patients at high risk requires attention as well.

Our study showed that the risks of amputation was only slightly increased in patients with canagliflozin treatment, but not in other subtypes of SGLT2i. It was proposed that partial inhibition of SGLT1 of canagliflozin might be associated with this subtype difference in inhibiting selectivity [23]. However, we did not observe increased risk of amputation and PAD in patients with sotagliflozin treatment, a dual inhibitor for SGLT1 and SGLT2 [24]. Another explanation may lie in the different amplitude of the body weight and blood pressure reduction among different SGLT2i subtypes. Although the pooled effect of all the SGLT2i subtypes could significantly reduce the body weight and blood pressure in patients with diabetes when compared with placebo treatment, our analysis showed that canagliflozin treatment led to the greatest weight reduction and the greatest BP reduction among all SGLT2i subtypes (Additional file 1: Table S3). It is assumed that the intensive amplitude of body weight and BP reduction caused by canagliflozin is more likely to exceed the autoregulation thresholds in patients at risk of lower limb complications while in patients with other subtypes of SGLT2i treatment, the alterations in hemodynamic status might be smoother and steadier. It might be a possible reason why the risk of amputation and PAD is only mildly increased in canagliflozin strata.

Actually, SGLT2i is a revolutionary antidiabetic agent which develops a novel treatment pattern for patients with diabetes. The clinical benefits from SGLT2i are much more than sorely improved glycemic control. SGLT2i confers more favorable pleiotropic effects on body weight, alanine aminotransferase and eGFR changes, potentially improving patients’ cardiometabolic disease risk when achieves hypoglycemic effects comparable to antidiabetic agents like dipeptidyl peptidase-4 inhibitor (DPP-4i) [25]. Moreover, studies showed that cardiovascular and renal benefits from SGLT2i treatment might be in part mediated by a state of fasting mimicry via several molecular mechanisms [26] and the regulation of autonomic nervous system [27]. SGLT2i may also ameliorate lipotoxic damage in stearate-treated myeloid angiogenic cells involved in atherosclerotic plaque vulnerability and/or thrombosis [28]. Together with the encouraging outcomes from CVOTs, SGLT2i is recommended as the first-line treatment for patients with arteriosclerotic cardiovascular disease, heart failure and chronic kidney disease in the latest American Diabetes Association Standards of Medical Care in Diabetes-2021, if their eGFRs meet the requirements [29].

In fact, SGLT2i is generally well tolerated in patients with diabetes. But the enhanced precaution action may further reduce the potential risk during the SGLT2i treatment course for patients susceptible to lower limb complications. Our study indicates that the amplitudes of reductions in body weight and blood pressure are associated with risks of lower limb complications, which calls for an additional attention to the alterations in body weight and blood pressure, two common indicators easy and feasible to monitor during the SGLT2i treatment. In studies in which canagliflozin was not included, SGLT2i were associated with lower risks of chronic heart failure and adverse lower limb events compared with DPP4i among patients with T2D and PAD in real-world practice [30]. It might be assumed that smooth and steady reductions in body weight and blood pressure, which do not exceed the autoregulation threshold, may not increase but reduce the risk of lower limb complications instead. In this situation, the hemostasis remains intact and meanwhile reductions in body weight and blood pressure will improve the insulin resistance, relieve the chronic inflammation and protect against vascular endothelial injuries. Therefore, monitoring the reductions in body weight and blood pressure will allow clinicians to adjust the SGLT2i regimens accordingly, which ensures the patients benefit from the SGLT2i treatment to the best extent without developing lower limb complications. The precaution action regarding body weight and blood pressure monitoring will promote a safe application of SGLT2i in the clinical settings.

There were some limitations in our studies. First, although there were adjudication committees for cardiovascular and renal endpoints in CVOTs and ROTs, and for important adverse events like urinary tract infection in efficacy and safety evaluation trials, there was no adjudication committees specially designed and set up for relevant lower limb complications in these RCTs. But as large event-driven RCTs, CVOTs and ROTs were basically with proper study design and reliable data assessments. The consistent results from CVOT and ROT strata enhance the creditability of our primary conclusion. Second, the definitions of PAD vary from trial to trial. To minimize the bias, we have unified the inclusion criteria of pre-specific PAD events and strictly stick to it during the data extraction. Moreover, several important data were not commonly reported in the published trials, including hematocrit, smoking status and previous PAD or amputation history. Actually, we did collect the available data about these alternative hemodynamic indicators and independent risk factors, and conducted the corresponding analyses. However, due to the limited data, we did not observe any significant association. Further analyses are still needed to validate and improve the hemodynamic hypothesis in the future.

## Conclusions

According to our meta-analysis, compared with non-SGLT2i users, the risks of amputation and PAD were slightly increased in patients with canagliflozin treatment. Greater reductions in body weight and blood pressure might be associated with the increased risk of lower limb complications in SGLT2i users. Monitoring body weight and blood pressure reduction might be an important risk precaution action for patients at high risk of lower limb complications during the SGLT2i treatment course. It might help the health care professionals achieve a safe application of SGLT2i in the clinical settings.

## Supplementary information

**Additional file 1: Figure S1.** Flowchart of included randomized controlled trials of SGLT2i treatment. **Figure S2.** The funnel plot of included randomized controlled trials of SGLT2i treatment in amputation analysis. **Figure S3.** The funnel plot of included randomized controlled trials of SGLT2i treatment in PAD analysis. **Figure S4.** The funnel plot of included randomized controlled trials of SGLT2i treatment in DF analysis. **Table S1.** The baseline characteristics of included trials of SGLT2i treatment. **Table S2.** The risk of bias for included trials of SGLT2i treatment. **Table S3.** Body weight and blood pressure reductions in patients with SGLT2i treatment.

## Data Availability

All data relevant to the study are included in the article or uploaded as additional file. No more additional data are available.

## Abbreviations

CI: Confidence interval
CVOT: Cardiovascular outcome trial
DF: Diabetic foot
DPP-4i: Dipeptidyl peptidase-4 inhibitor
eGFR: Estimated glomerular filtration rate
FDA: Food and Drug Administration
GLP-1RA: Glucagon-like peptide-1 receptor analog
OR: Odd ratio
PAD: Peripheral arterial disease
RCT: Randomized controlled trial
ROT: Renal outcome trial
SGLT2i: Sodium glucose co-transporter 2 inhibitor
T1D: Type 1 diabetes
T2D: Type 2 diabetes
WMD: Weighted mean difference
