# Correction to: SGLT2 inhibitors and lower limb complications: an updated meta-analysis

**DOI:** 10.1186/s12933-021-01306-6

**Published:** 2021-06-09

**Authors:** Chu Lin, Xingyun Zhu, Xiaoling Cai, Wenjia Yang, Fang Lv, Lin Nie, Linong Ji

**Affiliations:** 1 Department of Endocrinology and Metabolism, Peking University People’s Hospital, No.11 Xizhimen South Street, Xicheng District, Beijing, 100044 China; 2 Department of Endocrinology and Metabolism, Beijing Airport Hospital, Beijing, China

## Correction to: Cardiovasc Diabetol (2021) 20:91 10.1186/s12933-021-01276-9

Following publication of the original article [1], the authors regret the errors of the original data display in the forest plots, which has been corrected with this erratum.

For the analysis of amputation, in DAPA-CKD study, there should be 35 amputation events out of 2149 total events in SGLT2i treatment arm, and 39 amputation events out of 2149 total events in control treatment arm. And in DELIGHT study, there should be 1 amputation event out of 145 total events in SGLT2i treatment arm.

For the analysis of PAD and DF, there should be 573 total events in SGLT2i treatment arm in DEPICT-1 study, and there should be 419 total events in SGLT2i treatment arm in EMPA Barnett 2014, according to the data from *Clinicaltrial.gov*.

The data has been updated with in the new Fig. 1a and Fig. 1b. Some results from the sensitivity analyses were slightly changed and have been also updated in the new Table 1. The results of meta-regression remained unchanged in current reserved decimal digits. Such mild changes did not cause any substantial influence to the conclusion and clinical significance of our study.

**Fig. 1:**
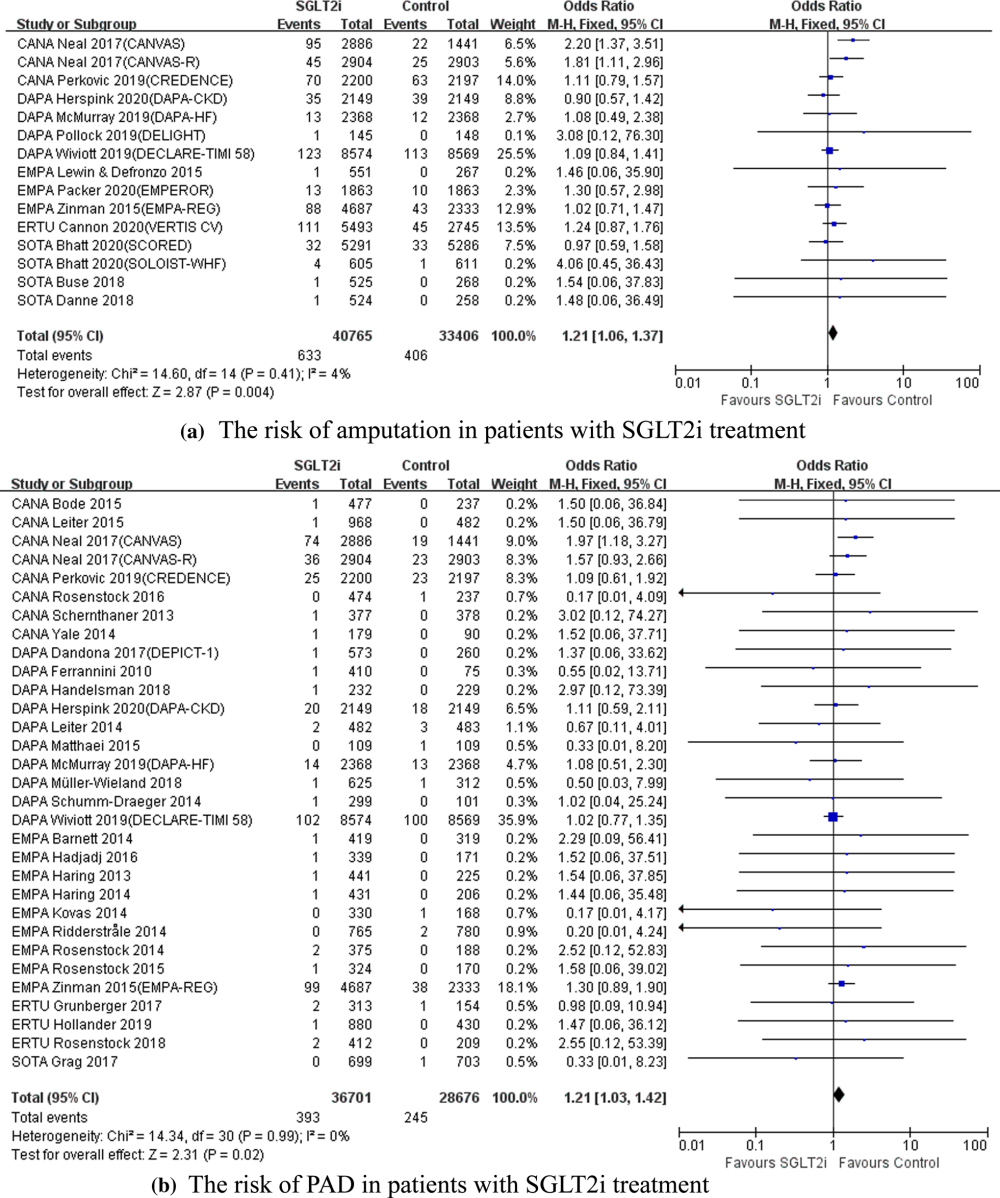
The risk of amputation and PAD in patients with SGLT2i treatment. **a** The risk of amputation in patients with SGLT2i treatment. **b** The risk of PAD in patients with SGLT2i treatment. *PAD* peripheral arterial disease, *SGLT2i* sodium glucose co-transporter 2 inhibitor

**Table 1:** Risk of amputation, PAD and DF events in patients with SGLT2i treatment

Subgroup	No. of participants (SGLT2i/control)	OR	95% CI	P value	I^2^ (%)
Risk of amputation by SGLT2i subtypes					
In total*	40,765/33,406	1.21	1.06, 1.37	0.004	4
Canagliflozin*	7990/6541	1.60	1.04, 2.46	0.03	67
Dapagliflozin	13,236/13,234	1.05	0.85, 1.30	0.66	0
Empagliflozin	7101/4463	1.07	0.76, 1.49	0.71	0
Ertugliflozin	5493/2745	1.24	0.87, 1.76	0.23	NA
Sotagliflozin	6945/6423	1.08	0.68, 1.70	0.75	0
Risk of amputation by study types					
CVOT and ROT*	39,020/32,465	1.20	1.06, 1.37	0.005	30
Efficacy and safety evaluation	1745/941	1.80	0.36, 8.95	0.47	0
Risk of amputation by population					
DM only*	34,715/27,194	1.24	1.08, 1.42	0.002	15
Including patients without DM	6380/6380	1.00	0.70, 1.43	1.00	0
Risk of amputation by control types					
Active agent	551/267	1.46	0.06, 35.90	0.82	NA
Placebo*	40,214/33,139	1.21	1.06, 1.37	0.004	11
Risk of amputation by study duration (weeks)					
< 26	145/148	3.08	0.12, 76.30	0.49	NA
26–52	2205/1404	2.34	0.58, 9.52	0.23	0
> 52*	38,415/31,854	1.20	1.05, 1.36	0.006	31
Risk of PAD by SGLT2i subtypes					
In total*	36,701/28,676	1.21	1.03, 1.42	0.02	0
Canagliflozin*	10,465/7965	1.53	1.14, 2.05	0.005	0
Dapagliflozin	15,821/14,655	1.02	0.81, 1.29	0.85	0
Empagliflozin	8111/4560	1.25	0.88, 1.78	0.21	0
Ertugliflozin	1605/793	1.49	0.30, 7.42	0.62	0
Sotagliflozin	699/703	0.33	0.01, 8.23	0.50	NA
Risk of PAD by study types					
CVOT and ROT*	25,768/21,960	1.24	1.05, 1.46	0.01	6
Efficacy and safety evaluation	10,933/6716	0.94	0.54, 1.63	0.82	0
Risk of PAD by population					
DM only*	32,184/24,159	1.22	1.03, 1.45	0.02	0
Including patients without DM	4517/4517	1.10	0.67, 1.79	0.71	0
Risk of PAD by control types					
Active agent	3847/2611	1.00	0.33, 3.06	1.00	0
Placebo*	32,854/26065	1.21	1.03, 1.43	0.02	0
Risk of PAD by study duration (weeks)					
< 26	5114/3162	0.90	0.43, 1.89	0.78	0
26–52	2855/1717	1.62	0.48, 5.52	0.44	0
> 52*	28,632/23,797	1.22	1.03, 1.44	0.02	0
Risk of DF by SGLT2i subtypes					
In total	32,043/25558	1.23	0.93, 1.63	0.15	0
Canagliflozin	9137/7113	1.55	0.94, 2.54	0.09	0
Dapagliflozin	14,586/13,806	1.20	0.79, 1.82	0.40	0
Empagliflozin	7127/4055	0.89	0.48, 1.65	0.71	0
Ertugliflozin	1193/584	1.48	0.15, 14.23	0.74	0
Risk of DF by study types					
CVOT and ROT	25,768/21,960	1.23	0.91, 1.66	0.17	0
Efficacy and safety evaluation	6275/3598	1.23	0.53, 2.84	0.63	0
Risk of DF by population					
DM only	27,526/21,041	1.27	0.95, 1.71	0.11	0
Including patients without DM	4517/4517	0.89	0.34, 2.31	0.81	0
Risk of DF by control types					
Active agent	4164/2459	1.53	0.44, 5.33	0.50	0
Placebo	27,879/23,099	1.22	0.91, 1.63	0.18	0
Risk of DF by study duration (weeks)					
< 26	1183/562	1.45	0.23, 9.22	0.69	0
26–52	3029/1606	1.45	0.42, 4.93	0.56	0
> 52	27,831/23,390	1.22	0.91, 1.63	0.19	0

*PAD* peripheral arterial disease, *SGLT2i* sodium glucose co-transporter 2 inhibitor, *DF* diabetic foot, *DM* diabetes mellitus, *CVOT* cardiovascular outcome trial, *ROT* renal outcome trial, *OR* odd ratio, *CI* confidence interval, *NA* not applicable

*P < 0.05

The contents in the abstract and main text have also been updated. All revisions are highlighted in bold fonts as follows.

In the result section of the abstract, the revision is shown as “The numbers of SGLT2i users versus non-SGLT2i users in the analyses of amputation, PAD and DF were **40,765/33,406**, **36,701/28,676** and **32,043/25,558** respectively”.

In the *Included studies* section of the main text, the revision is shown as “The numbers of SGLT2i users versus non-SGLT2i users in the analyses of amputation, PAD and DF were **40,765/33,406**, **36,701/28,676** and **32,043/25,558** respectively”.

In the *Risk of amputation, PAD and DF in patients with SGLT2i treatment* section of the main text, the revisions are shown as: (1) “Compared with non-SGLT2i users, the risk of amputation (**OR = 1.21**, **95% CI 1.06 to 1.37**, **P = 0.004**) (Fig. 1a) ……”; (2) “As for study population, the incidence of amputation (OR = 1.24, 95% CI 1.08 to 1.42, P = 0.002) and PAD (**OR = 1.22**, **95% CI 1.03 to 1.45**, **P = 0.02**) were significantly increased in SGLT2i users versus non-SGLT2i users……”; (3) “Moreover, the risk of amputation (**OR = 1.20**, **95% CI** **1.05 to 1.36**, **P = 0.006**) and the risk of PAD (OR = 1.22, 95% CI 1.03 to 1.44, P = 0.02) were significantly higher in RCTs with study duration longer than 52 weeks……”.
