# Supplementary material for: SGLT2 inhibitors and lower limb complications: an updated meta‐analysis
Source: Cardiovasc Diabetol. 2021 Apr 28;20:91. doi: 10.1186/s12933-021-01276-9 (PMC8082772; doi:10.1186/s12933-021-01276-9)
Supplement: Supplementary file 1 — Additional file 1: Figure S1. Flowchart of included randomized controlled trials of SGLT2i treatment. Figure S2. The funnel plot of included randomized controlled trials of SGLT2i treatment in amputation analysis. Figure S3. The funnel plot of included randomized controlled trials of SGLT2i treatment in PAD analysis. Figure S4. The funnel plot of included randomized controlled trials of SGLT2i treatment in DF analysis. Table S1. The baseline characteristics of included trials of SGLT2i treatment. Table S2. The risk of bias for included trials of SGLT2i treatment. Table S3. Body weight and blood pressure reductions in patients with SGLT2i treatment. [file 12933_2021_1276_MOESM1_ESM.docx]

**Additional file**

Additional figure

Figure S1. Flowchart of included randomized controlled trials of SGLT2i treatment

Figure S2. The funnel plot of included randomized controlled trials of SGLT2i treatment in amputation analysis

Figure S3. The funnel plot of included randomized controlled trials of SGLT2i treatment in PAD analysis

Figure S4. The funnel plot of included randomized controlled trials of SGLT2i treatment in DF analysis

Additional table

Table S1. The baseline characteristics of included trials of SGLT2i treatment

Table S2. The risk of bias for included trials of SGLT2i treatment

Table S3. Weight and blood pressure reduction in patients with SGLT2i treatments

Figure S1. Flowchart of included randomized controlled trials of SGLT2i treatment


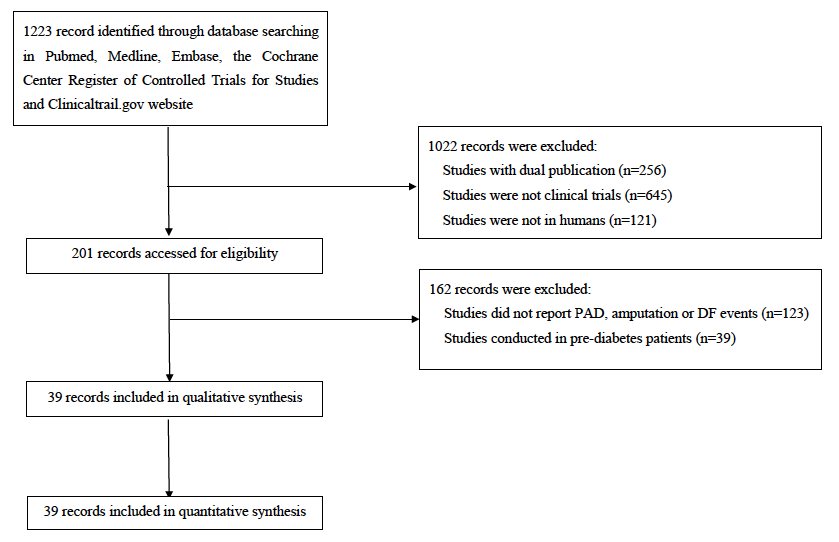


Figure S2. The funnel plot of included randomized controlled trials of SGLT2i treatment in amputation analysis


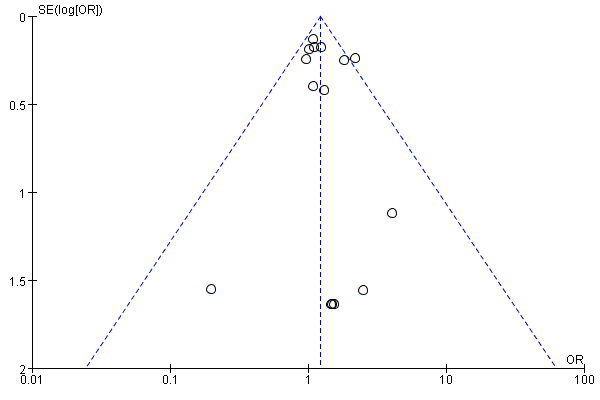


Figure S3. The funnel plot of included randomized controlled trials of SGLT2i treatment in PAD analysis


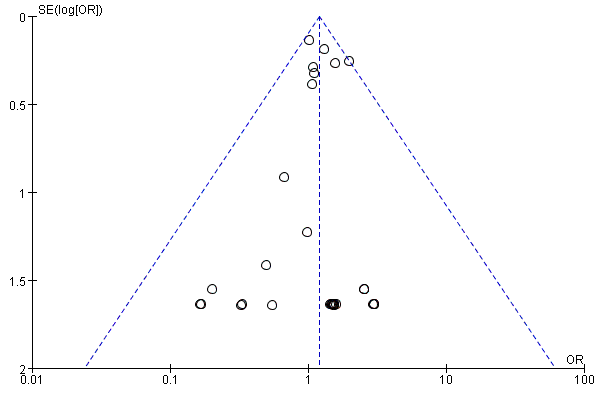


Figure S4. The funnel plot of included randomized controlled trials of SGLT2i treatment in DF analysis


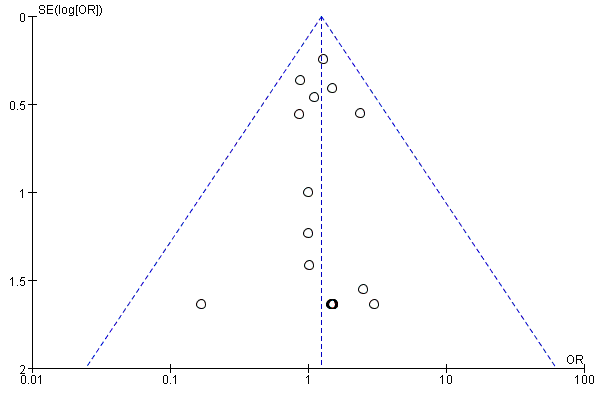


Table S1. The baseline characteristics of included trials of SGLT2i treatment

| Author, year | Study duration | Treatment group | No. of patients | Age (years) | Men (%) | BMI (kg/m^2^) | Weight (kg) | Duration of diabetes (years) | Baseline HbA1c (%) |
| --- | --- | --- | --- | --- | --- | --- | --- | --- | --- |
| Canagliflozin | | | | | | | | | |
| Bode, 2015^1^ | 104 weeks | Canagliflozin 300mg | 236 | 63.4±6.0 | 54.7 | 31.5±4.6 | 88.8±17.1 | 11.3±7.2 | 7.7±0.8 |
|  |  | Canagliflozin 100mg | 241 | 64.3±6.5 | 51.5 | 31.4±4.4 | 88.4±15.6 | 12.3±7.8 | 7.8±0.8 |
|  |  | Placebo | 237 | 63.2±6.2 | 60.3 | 31.8±4.7 | 91.1±17.5 | 11.4±7.3 | 7.8±0.8 |
| Leiter, 2015^2^ | 104 weeks | Canagliflozin 300mg | 485 | 55.8±9.2 | 49.7 | 31.2±5.4 | 86.6±19.5 | 6.7±5.5 | 7.8±0.8 |
|  |  | Canagliflozin 100mg | 483 | 56.4±9.5 | 52.2 | 31.0±5.3 | 86.9±20.1 | 6.5±5.5 | 7.8±0.8 |
|  |  | Glimepiride | 482 | 56.3±9.0 | 54.6 | 30.9±5.5 | 86.5±19.8 | 6.6±5.0 | 7.8±0.8 |
| Neal, 2017^3^  (CANVAS) | 188 weeks | Canagliflozin | 5644 | 63.2±8.3 | 64.9 | 31.9±5.9 | / | 13.5±7.7 | 8.2±0.9 |
|  |  | Placebo | 4231 | 63.4 ± 8.2 | 63.3 | 32.0 ± 6.0 | / | 13.7 ± 7.8 | 8.2±0.9 |
| Perkovic, 2019^4^ (CREDENCE) | 2.62 years | Canagliflozin | 2202 | 62.9±9.2 | 65.4 | 31.4±6.2 | / | 15.5±8.7 | 8.3±1.3 |
|  |  | Placebo | 2199 | 63.2±9.2 | 66.7 | 31.3±6.2 | / | 16.0±8.6 | 8.3±1.3 |
| Rosenstock, 2016^5^ | 26 weeks | Canagliflozin 300mg | 238 | 55.8±9.6 | 52.5 | 32.6±5.8 | 93.0±19.9 | 3.3±4.4 | 8.8±1.2 |
|  |  | Canagliflozin 100mg | 237 | 54.0±10.7 | 44.3 | 32.4±5.4 | 90.2±18.6 | 3.5±4.4 | 8.8±1.2 |
|  |  | Metformin | 237 | 55.2±9.8 | 48.9 | 33.0±6.0 | 92.1±20.1 | 3.3±4.5 | 8.8±1.2 |
| Schernthaner, 2013^6^ | 52 weeks | Canagliflozin 300mg | 377 | 56.6±9.6 | 54.9 | 31.5±6.9 | 87.4±23.2 | 9.4±6.1 | 8.1±0.9 |
|  |  | Sitagliptin 100 mg | 378 | 56.7±9.3 | 56.9 | 31.7±6.9 | 89.1±23.2 | 9.7±6.3 | 8.1±0.9 |
| Yale, 2014^7^ | 52 weeks | Canagliflozin 300mg | 89 | 67.9±8.2 | 53.9 | 33.4±6.5 | 90.2±18.1 | 17.0±7.8 | 8.0±0.8 |
|  |  | Canagliflozin 100mg | 90 | 69.5±8.2 | 64.4 | 32.4±5.5 | 90.5±18.4 | 15.6±7.4 | 7.9±0.9 |
|  |  | Placebo | 90 | 68.2±8.4 | 63.3 | 33.1±6.5 | 92.8±17.4 | 16.4±10.1 | 8.0±0.9 |
| Dapagliflozin | | | | | | | | | |
| Dandona, 2017^8^ | 24 weeks | Dapagliflozin 10mg + insulin | 259 | 42.7±14.1 | 50 | 28.1±5.1 | 82.0±17.3 | 19.9±11.1 | 8.52±0.64 |
| (DEPICT-1) |  | Dapagliflozin 5mg + insulin | 259 | 41.9±14.1 | 43 | 28.3±5.8 | 80.8±18.2 | 19.7±12.0 | 8.53±0.71 |
|  |  | Placebo + insulin | 260 | 42.7±13.6 | 51 | 28.6±5.2 | 84.3±18.3 | 21.2±12.2 | 8.53±0.67 |
| Ferrannini, 2010^9^ | 24 weeks | Dapagliflozin 10mg | 70 | 50.6±9.97 | 48.6 | 33.6±5.4 | 94.2±18.7 | 0.45 (0.10, 3.40) | 8.01±0.96 |
|  |  | Dapagliflozin 5mg | 64 | 52.6±10.9 | 48.4 | 31.9±4.8 | 87.6±17.1 | 0.25 (0.10, 1.40) | 7.86±0.94 |
|  |  | Dapagliflozin 2.5mg | 65 | 53.0±11.7 | 55.4 | 32.6±5.5 | 90.8±22.8 | 0.50 (0.10, 2.90) | 7.92±0.90 |
|  |  | Placebo | 75 | 52.7±10.3 | 41.3 | 32.5±5.5 | 90.8±22.8 | 0.50 (0.10, 3.40) | 7.84±0.87 |
| Handelsman, 2018^10^ | 52 weeks | Dapagliflozin + saxagliptin + metformin | 232 | 55.9±8.9 | 48.0 | 32.8±6.3 | / | 8.2±5.2 | 8.9±0.9 |
|  |  | Sitagliptin + metformin | 229 | 55.8±9.6 | 43.1 | 33.3±6.1 | / | 7.9±5.7 | 8.8±0.8 |
| Leiter, 2014^11^ | 52 weeks | Dapagliflozin 10mg | 480 | 63.9±7.6 | 66.9 | 33.0±5.3 | 94.5±17.8 | 13.5±8.2 | 8.0±0.8 |
|  |  | Placebo | 482 | 63.6±7.0 | 67.0 | 32.7±5.7 | 93.2±16.8 | 13.0±8.4 | 8.1±0.8 |
| Matthaei, 2015^12^ | 52 weeks | Dapagliflozin + metformin + sulphonylurea | 108 | 61.1±9.65 | 42.6 | 31.93±4.84 | 88.57±17.6 | ／ | 8.08±0.9 |
|  |  | Placebo + metformin + sulphonylurea | 108 | 60.9±9.24 | 55.6 | 32.02±4.58 | 90.07±16.2 | ／ | 8.24±0.9 |
| McMurray, 2019^13^  (DAPA-HF) | 18.2 months | Dapagliflozin 10mg | 2373 | 66.2±11.0 | 23.8 | 28.2±6.0 | / | / | / |
|  |  | Placebo | 2371 | 66.5±10.8 | 23 | 28.1±5.9 | / | / | / |
| Müller-Wieland, 2018^14^ | 52 weeks | Dapagliflozin + Saxagliptin + metformin | 312 | 59.2±7.9 | 60.9 | 32.5±5.1 | 95.3±17.4 | 7.3±5.9 | 8.3±0.7 |
|  |  | Dapagliflozin + metformin | 314 | 57.4±9.4 | 64.3 | 33.1±5.2 | 97.7±18.9 | 6.9±5.2 | 8.3±0.7 |
|  |  | Glimepiride + metformin | 313 | 58.6±8.4 | 66.5 | 33.0±5.1 | 97.3±17.9 | 6.7±5.1 | 8.3±0.8 |
| Pollock, 2019^15^  (DELIGHT) | 24 weeks | Dapagliflozin + saxagliptin | 155 | 64.0±9.2 | 71 | 30.81±5.4 | / | 18.43±8.1 | 8.20±1.0 |
|  |  | Dapagliflozin | 145 | 64.7±8.6 | 70 | 30.19±5.3 | / | 17.55±7.7 | 8.44±1.0 |
|  |  | Placebo | 148 | 64.7±8.5 | 71 | 30.34±5.6 | / | 17.71±9.5 | 8.57±1.2 |
| Schumm-Draeger, 2015^16^ | 16 weeks | Dapagliflozin 10mg + metformin | 99 | 58.5±9.8 | 49.5 | 32.35±5.01 | 90.58±15.9 | 5.45±4.1 | 7.71±0.71 |
|  |  | Dapagliflozin 5mg + metformin | 99 | 55.3±9.3 | 46.5 | 33.09±4.94 | 93.62±16.6 | 5.12±4.2 | 7.78±0.76 |
|  |  | Dapagliflozin 2.5mg + metformin | 100 | 58.3±9.0 | 37 | 33.16±5.16 | 92.49±18.6 | 4.80±3.9 | 7.77±0.75 |
|  |  | Placebo + metformin | 101 | 58.5±9.4 | 46.5 | 31.74±4.69 | 88.82±15.3 | 5.53±4.2 | 7.94±0.85 |
| Wiviott, 2019^17^ | 206 weeks | Dapagliflozin | 8582 | 63.9±6.8 | 63.1 | 32.1±6.0 | / | 11.0±5 | 8.3±1.2 |
| (DECLARE-TIMI 58) |  | Placebo | 8578 | 64.0±6.8 | 62.1 | 32.0±6.1 | / | 10.1±5 | 8.3±1.2 |
| Heerspink, 2020^18^ (DAPA-CKD) | 2.4 years | Dapagliflozin | 2152 | 61.8±12.1 | 67.1 | 29.4±6.0 | 81.5±20.5 | / | / |
|  |  | Placebo | 2152 | 61.9±12.1 | 66.7 | 29.6±6.3 | 82.0±20.9 | / | / |
| Empagliflozin |  |  |  |  |  |  |  |  |  |
| Barnett, 2014-CKD2^19^ | 52 weeks | Empagliflozin 25mg | 97 | 62.0±8.4 | 62.9 | 31.3±5.8 | 88.1±21.7 | / | 7.96±0.7 |
|  |  | Empagliflozin 10mg | 98 | 63.2±8.5 | 61.2 | 32.4±5.4 | 92.1±21.4 | / | 8.02±0.8 |
|  |  | Placebo | 95 | 62·6±8.1 | 58.9 | 30.8±5.6 | 86.0±20.0 | / | 8.09±0.8 |
| Barnett, 2014-CKD3^19^ | 52 weeks | Empagliflozin 25mg | 187 | 64.6±8.9 | 57.2 | 30.2±5.3 | 83.2±19.5 | / | 8.02±0.8 |
|  |  | Placebo | 187 | 65.1±8.2 | 56.7 | 30.3±5.3 | 82.5±18.0 | / | 8.09±0.8 |
| Barnett, 2014-CKD4^19^ | 52 weeks | Empagliflozin 25mg | 37 | 65.4±10.2 | 56.8 | 29.0±4.9 | 77.9±16.4 | / | 8.06±1.1 |
|  |  | Placebo | 37 | 62.9±11.9 | 51.4 | 31.8±6.0 | 84.1±21.1 | / | 8.16±1.0 |
| Hadjadj, 2016^20^ | 24 weeks | Empagliflozin 12.5mg + metformin 1000mg | 169 | 53.6±10.7 | 52.1 | 30.4±5.3 | 83.8±19.8 | / | 8.66±1.14 |
|  |  | Empagliflozin 5mg + metformin 1000mg | 167 | 52.3±11.3 | 59.3 | 30.5±5.0 | 83.0±19.1 | / | 8.65±1.23 |
|  |  | Empagliflozin 12.5mg + metformin 500mg | 165 | 51.0±10.7 | 63.6 | 30.2±5.2 | 82.9±18.7 | / | 8.84±1.31 |
|  |  | Empagliflozin 5mg + metformin 500mg | 161 | 52.2±11.7 | 60.2 | 30.1±5.3 | 82.3±19.2 | / | 8.65±1.23 |
|  |  | Metformin 1000mg | 164 | 51.6±10.8 | 56.1 | 30.5±5.9 | 83.7±20.1 | / | 8.58±1.13 |
|  |  | Metformin 500mg | 168 | 53.4±10.9 | 51.2 | 30.3±5.8 | 82.7±21.2 | / | 8.69±1.04 |
| Haring, 2013^21^ | 24 weeks | Empagliflozin 25mg | 216 | 57.4±9.3 | 53 | 28.3±5.5 | 77.5±18.8 | / | 8.10±0.83 |
|  |  | Empagliflozin 10mg | 225 | 57.0±9.2 | 30 | 28.3±5.4 | 77.1±18.3 | / | 8.07±0.81 |
|  |  | Placebo | 225 | 56.9±9.2 | 30 | 27.9±4.9 | 76.2±16.9 | / | 8.15±0.83 |
| Haring, 2014^22^ | 24 weeks | Empagliflozin 25mg | 213 | 55.6±10.2 | 56 | 29.7±5.7 | 82.2±19.3 | ／ | 7.86±0.87 |
|  |  | Empagliflozin 10mg | 217 | 55.5±9.9 | 58 | 29.1±5.5 | 81.6±18.5 | ／ | 7.94±0.79 |
|  |  | Placebo | 207 | 56.0±9.7 | 56 | 28.7±5.2 | 79.7±18.6 | ／ | 7.90±0.88 |
| Kovacs, 2014^23^ | 24 weeks | Empagliflozin 25mg | 168 | 54.2±8.9 | 50.6 | 29.1±5.5 | 78.9±19.9 | / | 8.1±0.82 |
|  |  | Empagliflozin 10mg | 165 | 54.7±9.9 | 50.3 | 29.2±5.6 | 78.0±19.1 | / | 8.1±0.89 |
|  |  | Placebo | 165 | 54.6±10.5 | 44.2 | 29.3±5.4 | 78.1±20.1 | / | 8.2±0.92 |
| Lewin, 2015^24^ | 24 weeks | Empagliflozin 25mg | 133 | 56.0±9.3 | 57.9 | 31.2±5.7 | 86.7±19.7 | / | 7.99±0.97 |
|  |  | Empagliflozin 10mg | 132 | 53.9±10.5 | 48.5 | 31.5±5.7 | 87.8±24.0 | / | 8.05±1.03 |
|  |  | Linagliptin 5mg | 133 | 53.8±11.5 | 56.4 | 31.9±5.9 | 89.5±20.1 | / | 8.05±0.89 |
| Defronzo, 2015^25^ | 52 weeks | Empagliflozin 25mg | 140 | 55.5±10.0 | 46.4 | 31.8±5.3 | 87.7±17.6 | / | 8.02±0.83 |
|  |  | Empagliflozin 10mg | 137 | 56.1±10.5 | 56.9 | 30.9±5.4 | 86.1±18.2 | / | 8.00±0.93 |
|  |  | Linagliptin 5mg | 128 | 56.2±10.0 | 50.0 | 30.6±5.4 | 85.0±18.3 | / | 8.02±0.90 |
| Ridderstråle, 2014^26^ | 104 weeks | Empagliflozin | 765 | 56.2±10.3 | 56 | 29.9±5.3 | 82.5±19.2 | / | 7.92±0.81 |
|  |  | Glimepiride | 780 | 55.7±10.4 | 54 | 30.3±5.3 | 83.0±19.2 | / | 7.92±0.86 |
| Rosenstock, 2014^27^ | 52 weeks | Empagliflozin 25mg + insulin | 189 | 58.0±9.4 | 44.0 | 35.0±4.0 | 95.9±17.3 | / | 8.29±0.7 |
|  |  | Empagliflozin 10mg + insulin | 186 | 56.7±8.7 | 52.0 | 34.7±3.8 | 96.7±17.9 | / | 8.39±0.7 |
|  |  | Placebo + insulin | 188 | 55.3±10.1 | 40.0 | 34.7±4.3 | 95.5±17.5 | / | 8.33±0.7 |
| Rosenstock, 2015^28^ | 78 weeks | Empagliflozin 25mg | 155 | 59.9±10.5 | 60.0 | 32.7±5.9 | 94.7±20.7 | / | 8.3±0.8 |
|  |  | Empagliflozin 10mg | 169 | 58.6±9.8 | 55.0 | 32.1±5.8 | 91.6±20.1 | / | 8.3±0.8 |
|  |  | Placebo | 170 | 58.1±9.4 | 53.0 | 31.8±6.0 | 90.5±22.5 | / | 8.2±0.8 |
| Zinman, 2015^29^ | 3.1 years | Empagliflozin 25mg | 2342 | 63.2±8.6 | 71.2 | 30.6±5.3 | 86.5±19.0 | / | 8.06±0.8 |
| (EMPA-REG) |  | Empagliflozin 10mg | 2345 | 63.0±8.6 | 70.5 | 30.6±5.2 | 85.9±18.8 | / | 8.07±0.8 |
|  |  | Placebo | 2333 | 63.2±8.8 | 72.0 | 30.7±5.2 | 86.6±19.1 | / | 8.08±0.8 |
| Packer, 2020^30^ | 16 months | Empagliflozin | 1863 | 67.2±10.8 | 76.5 | 28.0±5.5 | / | / | / |
| (EMPEROR-Reduced) |  | Placebo | 1867 | 66.5±11.2 | 75.6 | 27.8±5.3 | / | / | / |
| Ertugliflozin | | | | | | | | | |
| Grunberger, 2018^31^ | 52 weeks | Ertugliflozin 15mg | 155 | 67.5±8.5 | 48.4 | 31.7±5.3 | 85.8±17.4 | 14.5±8.5 | 8.2±0.9 |
|  |  | Ertugliflozin 5mg | 158 | 66.7±8.3 | 53.2 | 32.6±6.8 | 89.4±22.5 | 14.9±9.0 | 8.2±1.0 |
|  |  | Placebo | 154 | 67.5±8.9 | 46.8 | 33.2±6.1 | 90.4±18.9 | 13.1±8.1 | 8.1±0.9 |
| Hollander, 2019^32^ | 104 weeks | Ertugliflozin 15mg | 435 | 58.0±9.9 | 43.9 | 31.3±6.2 | 85.7±19.1 | 7.5±5.7 | 7.8±0.6 |
|  |  | Ertugliflozin 5mg | 445 | 58.7±9.8 | 51.0 | 31.7±5.6 | 88.0±19.0 | 7.3±5.7 | 7.8±0.6 |
|  |  | Glimepiride | 435 | 57.9±9.1 | 51.5 | 31.2±6.4 | 86.9±20.8 | 7.6±5.6 | 7.8±0.6 |
| Cannon, 2020^33^  (VERTIS CV) | 3.5 year | Ertugliflozin | 5499 | 64.4±8.1 | 70.3 | 31.9±5.4 | / | 12.9±8.3 | 8.2±1.0 |
|  |  | Placebo | 2747 | 64.4±8.0 | 69.3 | 32.0±5.5 | / | 13.1±8.4 | 8.2±0.9 |
| Rosenstock, 2018^34^ | 26 weeks | Ertugliflozin 15mg | 205 | 56.9±9.4 | 45.4 | 31.1±4.5 | 85.3±16.5 | 8.1±5.5 | 8.1±0.9 |
|  |  | Ertugliflozin 5mg | 207 | 56.6±8.1 | 46.9 | 30.8±4.8 | 84.8±17.2 | 7.9±6.1 | 8.1±0.9 |
|  |  | Placebo | 209 | 56.5±8.7 | 46.9 | 30.7±4.7 | 84.5±17.1 | 8.0±6.3 | 8.2±0.9 |
| Sotagliflozin | | | | | | | | | |
| Buse, 2016^35^ | 52 weeks | Sotagliflozin 400mg | 262 | 46.4±13.12 | 54.2 | 29.63±5.3 | 86.5±18.0 | 24.0±12.9 | 7.56±0.7 |
|  |  | Sotagliflozin 200mg | 263 | 46.6±13.48 | 52.1 | 29.81±5.7 | 87.0±18.5 | 25.0±13.2 | 7.61±0.7 |
|  |  | Placebo | 268 | 45.2±12.72 | 48.9 | 29.55±5.2 | 87.3±17.7 | 24.2±12.4 | 7.54±0.7 |
| Danne, 2008^36^ | 52 weeks | Sotagliflozin 400mg | 263 | 41.7±13.2 | 49.4 | 27.85±4.9 | 81.97±18.0 | 18.9±11.2 | 7.71±0.8 |
|  |  | Sotagliflozin 200mg | 261 | 42.3±13.6 | 46.7 | 27.97±5.3 | 81.93±17.4 | 18.2±10.8 | 7.74±0.8 |
|  |  | Placebo | 258 | 39.7±13.4 | 48.1 | 27.50±5.2 | 81.08±16.9 | 18.1±10.7 | 7.79±0.9 |
| Grag, 2017^37^ | 24 weeks | Sotagliflozin 400mg | 699 | 43.3±14.2 | 48.8 | 28.29±5.1 | 82.40±17.1 | 20.5±12.4 | 8.26±0.96 |
|  |  | Placebo | 703 | 42.4±14.0 | 51.8 | 28.10±5.2 | 81.55±17.0 | 19.6±12.1 | 8.21±0.92 |
| Bhatt, 2020^38^  (SCORED) | 16 months | Sotagliflozin 200-400mg | 5259 | 69 (63-74) | 55.7 | 31.9(28.1-36.2) | / | / | 8.3(7.6-9.3) |
|  |  | Placebo | 5292 | 69 (63-74) | 54.5 | 31.7(28.0-36.1) | / | / | 8.3(7.6-9.4) |
| Bhatt, 2020^39^  (SOLOIST-WHF) | 9 months | Sotagliflozin 200-400mg | 608 | 69 (63-76) | 67.4 | 30.4(26.3-34.3) | / | / | 7.1(6.4-8.3) |
|  |  | Placebo | 614 | 70 (64-76) | 65.1 | 31.1(27.3-34.5) | / | / | 7.2(6.4-8.2) |

Table S2. The risk of bias for included trials of SGLT2i treatment

| Author, year | Adequate randomization sequence generation | Adequate  allocation concealment | Blinding  of participants and caregivers | Binding of  outcome assessors  and adjudicators | Free of infrequent  missing outcome data | Free of selective outcome reporting | Free of other bias |
| --- | --- | --- | --- | --- | --- | --- | --- |
| Canagliflozin | | | | | | | |
| Bode, 2015^1^ | **Probably yes**  Randomized,  double-blind | **Probably yes**  Randomized, double-blind | **Definitely yes**  Double-blind  (participant,  investigator) | **Definitely yes** | **Probably no**  There were 15.4% (69/447) and 14.8% (35/237) patients in SGLT2i and control groups with missing outcome data, respectively; missing outcome data were generally balanced across treatment groups, with similar reasons for missing data across groups | **Definitely yes** | **Probably yes**  Generally balanced baseline  characteristics across groups |
| Leiter, 2015^2^ | **Probably yes**  Randomized,  double-blind | **Probably yes**  Randomized, double-blind | **Definitely yes**  Double-blind  (participant,  investigator) | **Definitely yes** | **Definitely no**  There were 31.2% (302/968) and 34.9% (168/482) patients in SGLT2i and control groups with missing outcome data, respectively; missing outcome data were generally balanced across treatment groups, with similar reasons for missing data across groups | **Definitely yes** | **Probably yes**  Generally balanced baseline  characteristics across groups |
| Neal, 2017^3^ | **Definitely yes**  Using an interactive web-based response system | **Probably yes**  Randomized, double-blind | **Definitely yes**  Double-blind  (participant,  investigator) | **Definitely yes** | **Definitely yes**  There were 4.2% (184/5795) and 3.9% (224/4347) patients in SGLT2i and control groups with missing outcome data, respectively; missing outcome data were generally balanced across treatment groups, with similar reasons for missing data across groups | **Definitely yes** | **Probably yes**  Generally balanced baseline  characteristics across groups |
| Perkovic, 2019^4^ | **Definitely yes**  Using an interactive web response system with a computer-generated randomization schedule | **Probably yes**  Randomized, double-blind | **Definitely yes**  Double-blind  (participant,  investigator) | **Definitely yes** | **Definitely yes**  There were 0.68% (15/2202) and 1.1% (25/2174) patients in SGLT2i and control groups with missing outcome data, respectively; missing outcome data were generally balanced across treatment groups, with similar reasons for missing data across groups | **Definitely yes** | **Probably yes**  Generally balanced baseline  characteristics across groups |
| Rosenstock, 2016^5^ | **Definitely yes**  Using a computer-generated randomization  schedule | **Probably yes**  Randomized, double-blind | **Definitely yes**  Double-blind  (participant,  investigator) | **Definitely yes** | **Probably no**  There were 10.1% (69/475) and 13.5% (32/237) patients in SGLT2i and control groups with missing outcome data, respectively; missing outcome data were generally balanced across treatment groups, with similar reasons for missing data across groups | **Definitely yes** | **Probably yes**  Generally balanced baseline  characteristics across groups |
| Schernthaner, 2013^6^ | **Definitely yes**  Using an Interactive Voice Response System/  Interactive Web Response System | **Probably yes**  Randomized, double-blind | **Definitely yes**  Double-blind  (participant,  investigator) | **Definitely yes** | **Definitely no**  There were 32.5% (123/378) and 44.4% (123/378) patients in SGLT2i and control groups with missing outcome data, respectively; missing outcome data were generally balanced across treatment groups, with similar reasons for missing data across groups | **Definitely yes** | **Probably yes**  Generally balanced baseline  characteristics across groups |
| Yale, 2014^7^ | **Probably yes**  Randomized | **Probably yes**  Randomized,  double-blind | **Probably yes**  Double-blind  (details not reported) | **Definitely yes** | **Probably no**  There were 20.1% (36/179) and 28.9% (26/90) patients in SGLT2i and control groups with missing outcome data, respectively; missing outcome data were generally balanced across treatment groups, with similar reasons for missing data across groups | **Definitely yes** | **Probably yes**  Generally balanced baseline  characteristics across groups |
| Dapagliflozin | | | | | | | |
| Dandona, 2017^8^ | **Definitely yes**  Using an  interactive voice response system | **Probably yes**  Randomized,  double-blind | **Probably yes**  Double-blind  (details not reported) | **Definitely yes** | **Probably no**  There were 9.1% (47/518) and 10.8% (28/260) patients in SGLT2i and control groups with missing outcome data, respectively; missing outcome data were generally balanced across treatment groups, with similar reasons for missing data across groups | **Definitely yes** | **Probably yes**  Generally balanced baseline  characteristics across groups |
| Ferrannini, 2010^9^ | **Probably yes**  Randomized | **Probably yes**  Randomized,  double-blind | **Probably yes**  Double-blind  (details not reported) | **Definitely yes** | **Probably no**  There were 15.7% (30/199) and 16.0% (12/75) patients in SGLT2i and control groups with missing outcome data, respectively; missing outcome data were generally balanced across treatment groups, with similar reasons for missing data across groups | **Definitely yes** | **Probably yes**  Generally balanced baseline  characteristics across groups |
| Handelsman, 2018^10^ | **Probably yes**  Randomized | **Probably yes**  Randomized,  double-blind | **Definitely yes**  Double-blind  (participant,  investigator) | **Definitely yes** | **Probably no**  There were 13.8% (32/232) and 5.2% (12/229) patients in SGLT2i and control groups with missing outcome data, respectively; missing outcome data were generally balanced across treatment groups, with similar reasons for missing data across groups | **Definitely yes** | **Probably yes**  Generally balanced baseline  characteristics across groups |

| Leiter, 2014^11^ | **Definitely yes**  Using a computer-generated random sequence via an interactive voice or web response system | **Probably yes**  Randomized,  double-blind | **Probably yes**  Double-blind  (details not reported) | **Definitely yes** | **Definitely no**  There were 31.2% (302/968) and 34.9% (168/482) patients in SGLT-2i and placebo groups with missing outcome data, respectively; missing outcome data were generally balanced across treatment groups, with similar reasons for missing data across groups | **Definitely yes** | **Probably yes**  Generally balanced baseline  characteristics across groups |
| --- | --- | --- | --- | --- | --- | --- | --- |

| Matthaei, 2015^12^ | **Definitely yes**  Using an interactive voice or web response system | **Probably yes**  Randomized, double-blind | **Definitely yes**  Double-blind  (participant,  investigator) | **Definitely yes** | **Probably no**  There were 8.3% (9/109) and 9.2% (10/109) patients in SGLT2i and control groups with missing outcome data, respectively; missing outcome data were generally balanced across treatment groups, with similar reasons for missing data across groups | **Definitely yes** | **Probably yes**  Generally balanced baseline  characteristics across groups |
| --- | --- | --- | --- | --- | --- | --- | --- |
| McMurray, 2019^13^ | **Definitely yes**  Using an interactive voice or web response system | **Probably yes**  Randomized | **Probably yes**  Randomized | **Definitely yes** | **Probably no**  There were 10.5% (249/2373) and 10.9% (258/2371) patients in SGLT2i and control groups with missing outcome data, respectively; missing outcome data were generally balanced across treatment groups, with similar reasons for missing data across groups | **Definitely yes** | **Probably yes**  Generally balanced baseline  characteristics across groups |
| Müller-Wieland, 2018^14^ | **Probably yes**  Randomized,  double-blind | **Probably yes**  Randomized,  double-blind | **Probably yes**  Double-blind  (details not reported) | **Definitely yes** | **Probably no**  There were 7.5% (47/626) and 8.0% (25/313) patients in SGLT2i and control groups with missing outcome data, respectively; missing outcome data were generally balanced across treatment groups, with similar reasons for missing data across groups | **Definitely yes** | **Probably yes**  Generally balanced baseline  characteristics across groups |
| Pollock, 2019^15^ | **Definitely yes**  using the sponsor’s interactive voice–web response  system | **Probably yes**  Randomized,  double-blind | **Definitely yes**  Double-blind  (participant,  investigator) | **Definitely yes** | **Probably no**  There were 6.7% (21/308) and 6.5% (10/153) patients in SGLT2i and control groups with missing outcome data, respectively; missing outcome data were generally balanced across treatment groups, with similar reasons for missing data across groups | **Definitely yes** | **Probably yes**  Generally balanced baseline  characteristics across groups |
| Schumm-Draeger, 2014^16^ | **Definitely yes**  Using an interactive web response system | **Probably yes**  Randomized,  double-blind | **Definitely yes**  Double-blind  (participant,  investigator) | **Definitely yes** | **Probably no**  There were 7.4% (22/299) and 7.9% (8/101) patients in SGLT2i and control groups with missing outcome data, respectively; missing outcome data were generally balanced across treatment groups, with similar reasons for missing data across groups | **Definitely yes** | **Probably yes**  Generally balanced baseline  characteristics across groups |
| Wiviott, 2019^17^ | **Definitely yes**  Using an interactive-voice/web response system | **Probably yes**  Randomized, double-blind | **Definitely yes**  Double-blind  (participant,  investigator) | **Definitely yes** | **Definitely yes**  There were 1.3% (109/8582) and 1.7% (145/8578) patients in SGLT2i and control groups with missing outcome data, respectively; missing outcome data were generally balanced across treatment groups, with similar reasons for missing data across groups | **Definitely yes** | **Probably yes**  Generally balanced baseline  characteristics across groups |
| Heerspink, 2020^18^ | **Definitely yes**  Using an interactive web response system | **Probably yes**  Randomized, double-blind | **Definitely yes**  Double-blind  (participant,  investigator) | **Definitely yes** | **Definitely yes**  There were 0.46% (10/2152) and 0.23% (5/2152) patients in SGLT2i and control groups with missing outcome data, respectively; missing outcome data were generally balanced across treatment groups, with similar reasons for missing data across groups | **Definitely yes** | **Probably yes**  Generally balanced baseline  characteristics across groups |
| Empagliflozin | | | | | | | |
| Barnett, 2014-CKD2^19^ | **Definitely yes**  Using a computer-generated random sequence | **Probably yes**  Randomized, double-blind | **Definitely yes**  Double-blind  (participant,  investigator) | **Definitely yes** | **Probably no**  There were 9.2% (18/195) and 8.2% (8/97) patients in SGLT2i and control groups with missing outcome data, respectively; missing outcome data were generally balanced across treatment groups, with similar reasons for missing data across groups | **Definitely yes** | **Probably yes**  Generally balanced baseline  characteristics across groups |
| Barnett, 2014-CKD3^19^ | **Definitely yes**  Using a computer-generated random sequence | **Probably yes**  Randomized, double-blind | **Definitely yes**  Double-blind  (participant,  investigator) | **Definitely yes** | **Probably no**  There were 12.2% (23/188) and 11.2% (21/187) patients in SGLT2i and control groups with missing outcome data, respectively; missing outcome data were generally balanced across treatment groups, with similar reasons for missing data across groups | **Definitely yes** | **Probably yes**  Generally balanced baseline  characteristics across groups |
| Barnett, 2014-CKD4^19^ | **Definitely yes**  Using a computer-generated random sequence | **Probably yes**  Randomized, double-blind | **Definitely yes**  Double-blind  (participant,  investigator) | **Definitely yes** | **Definitely no**  There were 29.7% (11/37) and 32.4% (12/37) patients in SGLT2i and control groups with missing outcome data, respectively; missing outcome data were generally balanced across treatment groups, with similar reasons for missing data across groups | **Definitely yes** | **Probably yes**  Generally balanced baseline  characteristics across groups |
| Hadjadj, 2016^20^ | **Definitely yes**  Using a  computer-generated random sequence  and an interactive voice and Web response  system. | **Probably yes**  Randomized, double-blind | **Definitely yes**  Double-blind  (participant,  investigator) | **Definitely yes** | **Probably no**  There were 7.8% (53/680) and 11.7% (40/341) patients in SGLT2i and control groups with missing outcome data, respectively; missing outcome data were generally balanced across treatment groups, with similar reasons for missing data across groups | **Definitely yes** | **Probably yes**  Generally balanced baseline  characteristics across groups |
| Haring, 2013^21^ | **Probably yes**  Randomized,  double-blind | **Probably yes**  Randomized,  double-blind | **Probably yes**  Double-blind  (details not reported) | **Definitely yes** | **Probably no**  There were 7.7% (34/441) and 10.7% (24/225) patients in SGLT2i and control groups with missing outcome data, respectively; missing outcome data were generally balanced across treatment groups, with similar reasons for missing data across groups | **Definitely yes** | **Probably yes**  Generally balanced baseline  characteristics across groups |
| Haring, 2014^22^ | **Definitely yes**  Using a  third-party interactive voice and web response  system, | **Probably yes**  Randomized,  double-blind | **Probably yes**  Double-blind  (details not reported) | **Definitely yes** | **Probably no**  There were 5.8% (25/430) and 10.1% (21/207) patients in SGLT2i and control groups with missing outcome data, respectively; missing outcome data were generally balanced across treatment groups, with similar reasons for missing data across groups | **Definitely yes** | **Probably yes**  Generally balanced baseline  characteristics across groups |
| Kovacs, 2014^23^ | **Definitely yes**  Using a computer-generated random sequence  and an interactive voice and web response system | **Probably yes**  Randomized,  double-blind | **Probably yes**  Double-blind  (details not reported) | **Definitely yes** | **Probably no**  There were 6.9% (23/333) and 10.9% (18/165) patients in SGLT2i and control groups with missing outcome data, respectively; missing outcome data were generally balanced across treatment groups, with similar reasons for missing data across groups | **Definitely yes** | **Probably yes**  Generally balanced baseline  characteristics across groups |
| Lewin, 2015^24^ | **Definitely yes**  Using a  third-party interactive voice and web response  system, | **Probably yes**  Randomized,  double-blind | **Probably yes**  Double-blind  (details not reported) | **Definitely yes** | **Probably no**  There were 17.0% (45/265) and 14.3% (19/133) patients in SGLT2i and control groups with missing outcome data, respectively; missing outcome data were generally balanced across treatment groups, with similar reasons for missing data across groups | **Definitely yes** | **Probably yes**  Generally balanced baseline  characteristics across groups |
| Defronzo, 2015^25^ | **Definitely yes**  Using a  third-party interactive voice and web response  system, | **Probably yes**  Randomized,  double-blind | **Probably yes**  Double-blind  (details not reported) | **Definitely yes** | **Probably no**  There were 13.7% (38/277) and 14.8% (19/128) patients in SGLT2i and control groups with missing outcome data, respectively; missing outcome data were generally balanced across treatment groups, with similar reasons for missing data across groups | **Definitely yes** | **Probably yes**  Generally balanced baseline  characteristics across groups |
| Ridderstråle, 2014^26^ | **Definitely yes**  Using an  interactive response system with a computer-generated  random sequence | **Probably yes**  Randomized, double-blind | **Definitely yes**  Double-blind  (participant,  investigator) | **Definitely yes** | **Probably no**  There were 16.9% (132/780) and 15.4% (117/760) patients in SGLT2i and control groups with missing outcome data, respectively; missing outcome data were generally balanced across treatment groups, with similar reasons for missing data across groups | **Definitely yes** | **Probably yes**  Generally balanced baseline  characteristics across groups |
| Rosenstock, 2014^27^ | **Definitely yes**  Using an interactive voice-and web-response system | **Probably yes**  Randomized, double-blind | **Definitely yes**  Double-blind  (participant,  investigator) | **Definitely yes** | **Probably no**  There were 15.6% (59/377) and 16.9% (32/189) patients in SGLT-2i and placebo groups with missing outcome data, respectively; missing outcome data were generally balanced across treatment groups, with similar reasons for missing data across groups | **Definitely yes** | **Probably yes**  Generally balanced baseline  characteristics across groups |
| Rosenstock, 2015^28^ | **Definitely yes**  Using a third-party interactive voice and web response system | **Probably yes**  Randomized, double-blind | **Definitely yes**  Double-blind  (participant,  investigator) | **Definitely yes** | **Definitely no**  There were 25.3% (82/324) and 30.6% (52/170) patients in SGLT-2i and placebo groups with missing outcome data, respectively; missing outcome data were generally balanced across treatment groups, with similar reasons for missing data across groups | **Definitely yes** | **Probably yes**  Generally balanced baseline  characteristics across groups |
| Zinman, 2015^29^ | **Definitely yes**  Using a computer-generated random-sequence and interactive voice-and web-response system | **Probably yes**  Randomized, double-blind | **Definitely yes**  Double-blind  (participant,  investigator) | **Definitely yes** | **Definitely yes**  There were 3.1% (144/4687) and 2.9% (67/2333) patients in active agent and placebo groups with missing outcome data, respectively; missing outcome data were generally balanced across treatment groups, with similar reasons for missing data across groups | **Definitely yes** | **Probably yes**  Generally balanced baseline  characteristics across groups |
| Packer, 2020^30^ | **Definitely yes**  Using a permuted block design with a computer pseudo-random number generator and an interactive response technology system | **Probably yes**  Randomized, double-blind | **Definitely yes**  Double-blind  (participant,  investigator) | **Definitely yes** | **Definitely yes**  There were 1.2% (22/1863) and 1.1% (20/1867) patients in active agent and placebo groups with missing outcome data, respectively; missing outcome data were generally balanced across treatment groups, with similar reasons for missing data across groups | **Definitely yes** | **Probably yes**  Generally balanced baseline  characteristics across groups |
| Ertugliflozin | | | | | | | |
| Grunberger, 2018^31^ | **Definitely yes**  Using an  interactive voice response system/integrated web  response system | **Probably yes**  Randomized, double-blind | **Definitely yes**  Double-blind  (participant,  investigator) | **Definitely yes** | **Probably no**  There were 17.5% (55/314) and 15.6% (24/154) patients in active agent and placebo groups with missing outcome data, respectively; missing outcome data were generally balanced across treatment groups, with similar reasons for missing data across groups | **Definitely yes** | **Probably yes**  Generally balanced baseline  characteristics across groups |
| Hollander, 2019^32^ | **Definitely yes**  Using a computer-  generated randomization schedule | **Probably yes**  Randomized, double-blind | **Definitely yes**  Double-blind  (participant,  investigator) | **Definitely yes** | **Definitely no**  There were 23.0% (202/880) and 24.8% (108/435) patients in active agent and placebo groups with missing outcome data, respectively; missing outcome data were generally balanced across treatment groups, with similar reasons for missing data across groups | **Definitely yes** | **Probably yes**  Generally balanced baseline  characteristics across groups |
| Cannon, 2020^33^ | **Probably yes**  Randomized,  double-blind | **Probably yes**  Randomized,  double-blind | **Probably yes**  Double-blind  (details not reported) | **Definitely yes** | **Probably no**  There were 12.0% and 13.0% patients in active agent and placebo groups with missing outcome data, respectively; missing outcome data were generally balanced across treatment groups, with similar reasons for missing data across groups | **Definitely yes** | **Probably yes**  Generally balanced baseline  characteristics across groups |
| Rosenstock, 2018^34^ | **Definitely yes**  Using a computer-generated randomization code based  on the method of random permuted blocks | **Probably yes**  Randomized, double-blind | **Definitely yes**  Double-blind  (participant,  investigator) | **Definitely yes** | **Probably no**  There were 4.9% (20/412) and 9.1% (19/209) patients in active agent and placebo groups with missing outcome data, respectively; missing outcome data were generally balanced across treatment groups, with similar reasons for missing data across groups | **Definitely yes** | **Probably yes**  Generally balanced baseline  characteristics across groups |
| Sotagliflozin | | | | | | | |
| Buse, 2018^35^ | **Probably yes**  Randomized,  double-blind | **Probably yes**  Randomized,  double-blind | **Probably yes**  Double-blind  (details not reported) | **Definitely yes** | **Probably no**  There were 9.3% (49/525) and 12.3% (33/268) patients in SGLT2i and control groups with missing outcome data, respectively; missing outcome data were generally balanced across treatment groups, with similar reasons for missing data across groups | **Definitely yes** | **Probably yes**  Generally balanced baseline  characteristics across groups |
| Danne, 2018^36^ | **Probably yes**  Randomized,  double-blind | **Probably yes**  Randomized,  double-blind | **Probably yes**  Double-blind  (details not reported) | **Definitely yes** | **Probably no**  There were 8.6% (45/524) and 8.5% (22/258) patients in SGLT2i and control groups with missing outcome data, respectively; missing outcome data were generally balanced across treatment groups, with similar reasons for missing data across groups | **Definitely yes** | **Probably yes**  Generally balanced baseline  characteristics across groups |
| Grag, 2017^37^ | **Probably yes**  Randomized,  double-blind | **Probably yes**  Randomized,  double-blind | **Probably yes**  Double-blind  (details not reported) | **Definitely yes** | **Probably no**  There were 13.6% (95/699) and 11.5% (81/703) patients in SGLT2i and control groups with missing outcome data respectively; missing outcome data were generally balanced across treatment groups, with similar reasons for missing data across groups | **Definitely yes** | **Probably yes**  Generally balanced baseline  characteristics across groups |
| Bhatt, 2020^38^  (SCORED) | **Probably yes**  Randomized,  double-blind | **Probably yes**  Randomized,  double-blind | **Definitely yes**  Double-blind  (participant,  investigator) | **Definitely yes** | **Definitely yes**  There were 1.1% (60/5292) and 1.5% (82/5292) patients in active agent and placebo groups with missing outcome data, respectively; missing outcome data were generally balanced across treatment groups, with similar reasons for missing data across groups | **Definitely yes** | **Probably yes**  Generally balanced baseline  characteristics across groups |
| Bhatt, 2020^39^  (SOLOIST-WHF) | **Definitely yes**  Using interactive-response technology | **Probably yes**  Randomized,  double-blind | **Definitely yes**  Double-blind  (participant,  investigator) | **Definitely yes** | **Definitely yes**  There were 3.3% (20/608) and 3.7% (23/614) patients in active agent and placebo groups with missing outcome data, respectively; missing outcome data were generally balanced across treatment groups, with similar reasons for missing data across groups | **Definitely yes** | **Probably yes**  Generally balanced baseline  characteristics across groups |

Table S3. Weight and blood pressure reduction in patients with SGLT2i treatments

|  | Weight reduction | | SBP reduction | | DBP reduction | |
| --- | --- | --- | --- | --- | --- | --- |
| Treatment type | WMD (kg) | 95%CI | WMD (mmHg) | 95%CI | WMD (mmHg) | 95%CI |
| SGLT2i in total | -2.28 | -2.50, -2.06 | -3.51 | -3.82, -3.20 | -1.67 | -1.88, -1.46 |
| *Canagliflozin* | -2.80* | -3.26, -2.35 | -4.37*^†^ | -5.10, -3.65 | -1.58 | -1.97, -1.19 |
| *Dapagliflozin* | -2.17 | -2.55, -1.79 | -2.80 | -3.35, -2.25 | -1.29 | -1.87, -0.71 |
| *Empagliflozin* | -2.28 | -2.61, -1.95 | -3.44 | -4.10, -2.78 | -1.92 | -2.37, -1.48 |
| *Ertugliflozin* | -2.36 | -2.69, -2.04 | -3.47 | -4.40, -3.49 | -1.80 | -1.94, -1.67 |
| *Sotagliflozin* | -2.78 | -3.86, -1.71 | -3.19 | -3.86, -2.52 | -1.30 | -1.85, -0.75 |

*Compared with dapagliflozin, P<0.05; †Compared with sotagliflozin, P<0.05. BP, blood pressure; SBP, systolic blood pressure; DBP, dilated blood pressure; SGLT2i, sodium glucose co-transporter 2 inhibitor; WMD, weighted mean difference; CI, confidence interval.

Reference

1. Bode B, K. Stenlöf, Harris S, et al. Long‐term efficacy and safety of canagliflozin over 104 weeks in patients aged 55–80 years with type 2 diabetes. Diabetes Obes Metab. 2015;17(3):294-303. DOI: 10.1111/dom.12428.
2. Leiter L A, Yoon K H, Arias P, et al. Canagliflozin Provides Durable Glycemic Improvements and Body Weight Reduction Over 104 Weeks Versus Glimepiride in Patients with Type 2 Diabetes on Metformin: A Randomized, Double-Blind, Phase 3 Study. Diabetes Care. 2015, 38(3):355-364. DOI: 10.2337/dc13-2762.
3. Neal B, Perkovic V, Mahaffey K, et al. Canagliflozin and cardiovascular and renal events in type 2 diabetes. N Engl J Med. 2017; 377:644-657. DOI: 10.1056/NEJMoa1611925.
4. Perkovic V, Jardine M J, Neal B, et al. Canagliflozin and Renal Outcomes in Type 2 Diabetes and Nephropathy. N Engl J Med. 2019; 380:2295-2306. DOI: 10.1056/NEJMoa1811744
5. Rosenstock J, Chuck L, Manuel González-Ortiz, et al. Initial Combination Therapy With Canagliflozin Plus Metformin Versus Each Component as Monotherapy for Drug-Naive Type 2 Diabetes. Diabetes care. 2016, 39(3):353-362. DOI: 10.2337/dc15-1736
6. Schernthaner G, Gross J L, Rosenstock J, et al. Canagliflozin compared with sitagliptin for patients with type 2 diabetes who do not have adequate glycemic control with metformin plus sulfonylurea: a 52-week randomized trial. Diabetes care. 2013, 36(9):2508-2515. DOI: 10.2337/dc12-2491.
7. Yale J F, Bakris G, Carious B, et al. Efficacy and Safety of Canagliflozin Over 52 Weeks in Patients With Type 2 Diabetes Mellitus and Chronic Kidney Disease. Diabetes Obes Metab. 2014;16(10):1016-1027. DOI: 10.1111/dom.12348.
8. Dandona P, Mathieu C, Phillip M, et al. Efficacy and safety of dapagliflozin in patients with inadequately controlled type 1 diabetes (DEPICT-1) 24 week results from a multicentre, double-blind, phase 3, randomised controlled trial. Lancet Diabetes Endocrinol. 2017, 5(11):864-876. DOI: 10.1016/S2213-8587(17)30308-X.
9. Ferrannini E , Ramos S J , Salsali A , et al. Dapagliflozin Monotherapy in Type 2 Diabetic Patients With Inadequate Glycemic Control by Diet and Exercise A randomized, double-blind, placebo-controlled, phase 3 trial. Diabetes care. 2010, 33(10):2217-2224. DOI: 10.2337/dc10-0612.
10. Handelsman Y, Mathieu C, Del Prato S, et al. Sustained 52-week efficacy and safety of triple therapy with dapagliflozin plus saxagliptin versus dual therapy with sitagliptin added to metformin in patients with uncontrolled type 2 diabetes. Diabetes Obes Metab. 2019;21(4):883-892. DOI: 10.1111/dom.13594.
11. Leiter L A, Cefalu W T, De Bruin T W A, et al. Dapagliflozin Added to Usual Care in Individuals with Type 2 Diabetes Mellitus with Preexisting Cardiovascular Disease: A 24-Week, Multicenter, Randomized, Double-Blind, Placebo-Controlled Study with a 28-Week Extension. J Am Geriatr Soc. 2014;62(7):1252-1262. DOI: 10.1111/jgs.12881.
12. Matthaei S, Bowering K, Rohwedder K, et al. Durability and tolerability of dapagliflozin over 52 weeks as add-on to metformin and sulphonylurea in type 2 diabetes. Diabetes Obes Metab. 2015;17(11):1075-1084. DOI: 10.1111/dom.12543.
13. Mcmurray J J V, Solomon S D, Inzucchi S E, et al. Dapagliflozin in Patients with Heart Failure and Reduced Ejection Fraction. N Engl J Med 2019; 381:1995-2008. DOI: 10.1056/NEJMoa1911303.
14. Müller-Wieland Dirk, Monika K, Katarzyna C, et al. Efficacy and Safety of Dapagliflozin or Dapagliflozin Plus Saxagliptin Versus Glimepiride as Add-on to Metformin in Patients With Type 2 Diabetes. Diabetes Obes Metab. 2018;20(11):2598-2607. DOI: 10.1111/dom.13437.
15. Pollock C, Stefánsson B, Reyner D, et al. Albuminuria-lowering effect of dapagliflozin alone and in combination with saxagliptin and effect of dapagliflozin and saxagliptin on glycaemic control in patients with type 2 diabetes and chronic kidney disease (DELIGHT): a randomised, double-blind, placebo-controlled trial. Lancet Diabetes Endocrinol. 2017, 5(11):864-876. DOI: 10.1016/S2213-8587(19)30086-5.
16. Schumm-Draeger P M, Burgess L, Korányi L, et al. Twice-daily dapagliflozin co-administered with metformin in type 2 diabetes: a 16-week randomized, placebo-controlled clinical trial. Diabetes Obes Metab. 2015;17(1):42-51. DOI: 10.1111/dom.12387.
17. Wiviott S, Raz I, Bonaca M P, et al. Dapagliflozin and Cardiovascular Outcomes in Type 2 Diabetes. N Engl J Med 2019; 380:347-357. DOI: 10.1056/NEJMoa1812389.
18. Heerspink H JL, Stefánsson B V, Correa-Rotter R, et al. Dapagliflozin in Patients with Chronic Kidney Disease. N Engl J Med. 2020;383(15):1436-1446.
19. Barnett, A H, Mithal A, Manassie J, et al. Efficacy and safety of empagliflozin added to existing antidiabetes treatment in patients with type 2 diabetes and chronic kidney disease: a randomised, double-blind, placebo-controlled trial. Lancet Diabetes Endocrinol. 2014;2(5):369-84. DOI: 10.1016/S2213-8587(13)70208-0.
20. Hadjadj S, Rosentsock J, Meinicke T, et al. Initial Combination of Empagliflozin and Metformin in Patients With Type 2 Diabetes. Diabetes Care. 2016; 39(10): 1718-1728. DOI: 10.2337/dc16-0522.
21. Häring H-U, Merker L, Seewaldt-Becker E, et al. Empagliflozin as add-on to metformin plus sulfonylurea in patients with type 2 diabetes. Diabetes Care. 2013;36(11):3396-3404. DOI: 10.2337/dc12-2673.
22. Häring H-U, Merker L, Seewaldt-Becker E, et al. Empaglif lozin as Add-On to Metformin in Patients With Type 2 Diabetes: A 24-Week, Randomized, Double-Blind, Placebo-Controlled Trial. Diabetes Care 2014;37(6):1650–1659. DOI: 10.2337/dc13-2105.
23. Kovacs C S, Seshiah V, Swallow R, et al. Empagliflozin improves glycaemic and weight control as add-on therapy to pioglitazone or pioglitazone plus metformin in patients with type 2 diabetes: a 24-week, randomized, placebo-controlled trial. Diabetes Obes Metab. 2014;16(2):147-158. DOI: 10.1111/dom.12188.
24. Lewin A, Defronzo R A, Patel S, et al. Initial Combination of Empagliflozin and Linagliptin in Subjects With Type 2 Diabetes. Diabetes Care. Diabetes Care. 2015;38(3):394-402. DOI: 10.2337/dc14-2365.
25. Defronzo R A, Lewin A, Patel S, et al. Combination of empagliflozin and linagliptin as second-line therapy in subjects with type 2 diabetes inadequately controlled on metformin. Diabetes Care. 2015;38(3):384-393. DOI: 10.2337/dc14-2364.
26. Ridderstråle M, Andersen K R, Zeller C, et al. Comparison of Empagliflozin and Glimepiride as Add-On to Metformin in Patients With Type 2 Diabetes: A 104-week Randomised, Active-Controlled, Double-Blind, Phase 3 Trial. Lancet Diabetes Endocrinol. 2014;2(9):691-700. DOI: 10.1016/S2213-8587(14)70120-2.
27. Rosenstock J, Jelaska A, Frappin G, et al. Improved Glucose Control With Weight Loss, Lower Insulin Doses, and No Increased Hypoglycemia With Empagliflozin Added to Titrated Multiple Daily Injections of Insulin in Obese Inadequately Controlled Type 2 Diabetes. Diabetes Care. 2014, 37(7):1815-1823. DOI: 10.2337/dc13-3055.
28. Rosenstock J, Jelaska A, Zeller C, et al. Impact of empagliflozin added on to basal insulin in type 2 diabetes inadequately controlled on basal insulin: a 78-week randomized, double-blind, placebo-controlled trial. Diabetes Obes Metab. 2015 ;17(10):936-948. DOI: 10.1111/dom.12503.
29. Zinman B, Wanner C, Lachin J M, et al. Empagliflozin, Cardiovascular Outcomes, and Mortality in Type 2 Diabetes. N Engl J Med. 2015;26;373(22):2117-2128. DOI: 10.1056/NEJMoa1504720.
30. Packer M, Anker S D, Butler J, et al. Cardiovascular and Renal Outcomes with Empagliflozin in Heart Failure. N Engl J Med. 2020;383(15):1413-1424.
31. Grunberger G, Camp S, Johnson J, et al. Ertugliflozin in Patients with Stage 3 Chronic Kidney Disease and Type 2 Diabetes Mellitus: The VERTIS RENAL Randomized Study. Diabetes Ther. 2018;9(1):49-66. DOI: 10.1007/s13300-017-0337-5.
32. Hollander P, Hill J, Johnson J, et al. Results of VERTIS SU extension study: safety and efficacy of ertugliflozin treatment over 104 weeks compared to glimepiride in patients with type 2 diabetes mellitus inadequately controlled on metformin. Curr Med Res Opin. 2019 ;35(8):1335-1343. DOI: 10.1080/03007995.2019.1583450.
33. Cannon C P , Pratley R , Dagogo-Jack S , et al. Cardiovascular Outcomes with Ertugliflozin in Type 2 Diabetes. N Engl J Med. 2020;383(15):1425-1435.
34. Rosenstock J, Frias J, Dénes Páll, et al. Effect of ertugliflozin on glucose control, body weight, blood pressure and bone density in type 2 diabetes mellitus inadequately controlled on metformin monotherapy (VERTIS MET). Diabetes Obes Metab. 2018;20(3):520-529. DOI: 10.1111/dom.13103.
35. Buse J B, Garg S K, Julio R, et al. Sotagliflozin in Combination With Optimized Insulin Therapy in Adults With Type 1 Diabetes: The North American inTandem1 Study. Diabetes Care. 2018;41(9):1970-1980. DOI: 10.2337/dc18-0343.
36. Danne T, Cariou B, Banks P, et al. HbA 1c and Hypoglycemia Reductions at 24 and 52 Weeks With Sotagliflozin in Combination With Insulin in Adults With Type 1 Diabetes: The European inTandem2 Study. Diabetes Care. 2018;41(9):1981-1990. DOI: 10.2337/dc18-0342.
37. Garg S K, Henry R R, Banks P, et al. Effects of Sotagliflozin Added to Insulin in Patients With Type 1 Diabetes. N Engl J Med. 2017;377(24):2337-2348. DOI: 10.1056/NEJMoa1708337.
38. Bhatt DL, Szarek M, Pitt B, et al. Sotagliflozin in Patients with Diabetes and Chronic Kidney Disease. N Engl J Med. 2020 Nov 16. DOI: 10.1056/NEJMoa2030186.
39. Bhatt DL, Szarek M, Steg PG, et al. Sotagliflozin in Patients with Diabetes and Recent Worsening Heart Failure. N Engl J Med. 2020 Nov 16. DOI: 10.1056/NEJMoa2030183.
